# PepLand: a large-scale pre-trained peptide representation model for a comprehensive landscape of both canonical and non-canonical amino acids

**DOI:** 10.1093/bib/bbaf367

**Published:** 2025-08-01

**Authors:** Ruochi Zhang, Haoran Wu, Chang Liu, Qian Yang, Yuting Xiu, Kewei Li, Ningning Chen, Yu Wang, Yan Wang, Xin Gao, Fengfeng Zhou

**Affiliations:** Key Laboratory of Symbolic Computation and Knowledge Engineering of Ministry of Education, Jilin University, Changchun, Jilin 130012, China; School of Artificial Intelligence, Jilin University, Changchun 130012, China; Syneron Technology, Guangzhou 510700, China; Syneron Technology, Guangzhou 510700, China; Beijing Life Science Academy, Beijing 102209, China; Key Laboratory of Symbolic Computation and Knowledge Engineering of Ministry of Education, Jilin University, Changchun, Jilin 130012, China; College of Computer Science and Technology Jilin University, Changchun, Jilin 130012, China; Syneron Technology, Guangzhou 510700, China; Key Laboratory of Symbolic Computation and Knowledge Engineering of Ministry of Education, Jilin University, Changchun, Jilin 130012, China; College of Computer Science and Technology Jilin University, Changchun, Jilin 130012, China; Syneron Technology, Guangzhou 510700, China; Syneron Technology, Guangzhou 510700, China; Key Laboratory of Symbolic Computation and Knowledge Engineering of Ministry of Education, Jilin University, Changchun, Jilin 130012, China; School of Artificial Intelligence, Jilin University, Changchun 130012, China; College of Computer Science and Technology Jilin University, Changchun, Jilin 130012, China; Computational Bioscience Research Center, King Abdullah University of Science and Technology (KAUST) Thuwal, Thuwal 23955, Saudi Arabia; Computer Science Program, Computer, Electrical and Mathematical Sciences and Engineering Division, King Abdullah University of Science and Technology (KAUST) Thuwal, Thuwal 23955, Saudi Arabia; Key Laboratory of Symbolic Computation and Knowledge Engineering of Ministry of Education, Jilin University, Changchun, Jilin 130012, China; College of Computer Science and Technology Jilin University, Changchun, Jilin 130012, China

**Keywords:** peptide representation, peptide with non-canonical amino acids, pre-trained peptide model

## Abstract

The recent interest in peptides incorporating non-canonical amino acids has surged within the scientific community, driven by their enhanced stability and resistance to proteolytic degradation. These so-called non-canonical peptides offer significant potential for modifying biological, pharmacological, and physiochemical characteristics in both native and synthetic contexts. Despite their advantages, there remains a notable gap in the availability of an efficient pre-trained model capable of effectively capturing feature representations from such intricate peptide sequences. This study herein introduces PepLand, a novel pre-training framework designed for the comprehensive representation and analysis of peptides, encompassing both canonical and non-canonical amino acids. PepLand leverages a general-purpose multi-view heterogeneous graph neural network to unveil the subtle structural representations of peptides. Our empirical evaluations demonstrate PepLand’s proficiency in a range of peptide property prediction tasks, including cell penetrability, solubility, and protein–peptide binding affinity. These rigorous assessments affirm PepLand’s superior capability in discerning critical representations of peptides with both canonical and non-canonical amino acids, and provide a robust foundation for transformative advances in peptide-focused pharmaceutical research. We have made the entire source code and datasets available at http://www.healthinformaticslab.org/supp/resources.php or https://github.com/zhangruochi/PepLand

## Introduction

Natural peptides have increasingly served as a cornerstone of drug development, owing to their inherent therapeutic properties [1]. Their high specificity, potent efficacy, and relatively high safety contribute significantly towards their growing appeal in the pharmaceutical landscape [2]. Non-canonical amino acids serve as foundational elements for the development of peptide-based materials and therapeutic agents, and facilitate chemists in overcoming the structural and functional constraints traditionally associated with the limited repertoire of canonical amino acids [3]. For instances, cyclic peptides with both canonical and non-canonical amino acids are linked at distant points to create macrocyclic structures [4], they exhibit diverse biological activities, including acting as signaling agents in complex biological processes [5].

Deep learning techniques are being extensively explored for the prediction tasks of both peptide properties and peptide-entity interactions, where the entity can be proteins or diseases [6, 7]. These applications further accelerate the pace of drug discovery and development processes, but also present unique challenges. The existing models, such as the Evolutionary Scale Modeling (ESM) [8] and ProteinBert [9], leverage amino acid sequences to learn and predict co-evolutionary information embedded in protein sequences. These models have showcased noteworthy success in protein-related prediction tasks, but their efficacy is compromised when dealing with peptides. This shortcoming arises from the fundamental differences between proteins and peptides. Peptides generally have shorter lengths compared to proteins, hence their data distribution does not align well with the existing models that have been trained predominantly on protein sequences.

Another critical limitation of the existing pre-trained models such as ESM is their incapacity to effectively handle non-canonical amino acids, where are frequently used to enhance the pharmaceutical properties of peptides [9, 10]. Peptides may also be encoded by the pre-trained models of small molecules like ChemBERTa [11]. The data distributions of peptides and small molecules are substantial different, e.g. the length of a peptide is usually 5 to 10 times longer than that of a small molecule in the Simplified Molecular-Input Line-Entry System encoding [12].

Fragment-based methods, such as those seen in DeepFrag [13] and other molecule generation algorithms [14–16], have shown great promise in the small molecule domain by facilitating lead optimization and the identification of chemical fragments with improved binding affinity. These methods typically focus on the decomposition of molecules into functional motifs with drug targeting specificities. However, their application to synthetic peptides, which often contain repetitive units like amide bonds and various chemical modifications, remains underexplored. This presents a significant opportunity for innovation in the peptide field, where the complexity and variability of peptide structures pose unique challenges not typically encountered in small molecule research.

This study introduces PepLand, a large-scale pre-trained peptide representation model, specifically developed to bridge the current gap in the field. Initially, our approach involves encoding peptides composed of both canonical and non-canonical amino acids using molecular structures. A novel fragmentation algorithm is then employed to discern the optimally granular elements within these peptide structures. The PepLand framework is equipped with this distinctive capability to handle both canonical and non-canonical amino acids concurrently. It integrates granular components (fragments) and the atom view within a multi-view heterogeneous graph neural network, and facilitates a consensus representation of peptides across various levels of granularity.

To overcome the challenges posed by the relatively limited data on non-canonical amino acids, PepLand employs a two-step training strategy, which enhances its learning efficiency from datasets pertaining to both amino acid types. Our experiments validate that PepLand sets a new benchmark in diverse peptide property prediction tasks, including cell penetrability, solubility, and protein binding affinity. Our additional analysis confirms the pivotal role of the fragmentation approach in downstream peptide property prediction tasks. The applicability of PepLand is further evidenced through its successful deployment in two pharmaceutical contexts: predicting the binding affinity of cyclic peptides and assessing peptide synthesizability.

## Material and Methods

### Training datasets

This study utilizes a two-stage pre-training strategy. The training dataset in the first stage consists of 7 924 509 samples with both canonical and non-canonical amino acids from the comprehensive UniProt database [17]. We call the peptides with non-canonical amino acids as ‘non-canonical peptides’, and those with only canonical amino acids as ‘canonical peptides’. We have filtered out the sequences exceeding 30 residues in length, and curate a refined subset of short sequences with ~8 million entries specifically tailored to the needs of this study.

The second pre-training stage focuses on peptides with non-canonical amino acids. We incorporate CycPeptMPDB [18], a specialized dataset comprising 7334 cell-penetrating peptides with non-canonical amino acids. An additional set of high-quality non-canonical peptides are collected from the RCSB Protein Data Bank (PDB) database [19]. The final dataset for the second pre-training stage comprises 8977 non-canonical peptides, providing a diverse and representative training resource for our model.

### Evaluating datasets

We conduct a rigorous evaluation of our pre-trained PepLand model through the curated array of datasets, spanning the prediction tasks of cell penetration ability, solubility, and protein-peptide binding affinity. Both canonical and non-canonical peptides are covered. We collected and curated these evaluation datasets from various databases and public literatures. To facilitate further research in the field of peptides, we have made these datasets publicly available together with our source code. The details of the datasets are given in the section ‘Evaluating Datasets’ in the Supplementary Materials.

### Overall Workflow for PepLand

This study proposes PepLand, a multi-view heterogeneous graph structure that integrates three distinct perspectives: the atom view, the junction view, and the fragment view. This graph facilitates information exchange between nodes and edges across views through an advanced message passing mechanism. The proposed heterogeneous graph framework represents atoms as nodes, and their interactions as edges, comprising the atom view. This view is specifically tailored to encapsulate atomic level details of peptides.

Our focus on non-canonical amino acid modifications is driven by their established roles in enhancing peptide stability and altering biological properties. For instance, modifications of D-amino acids are known to augment peptide stability, while alterations with *α*–amino acids can stimulate both biological stability and resistance to degradation [20]. Specific modifications of prolines are particularly noteworthy for their applications in conformational studies and in modulating the properties of both naturally occurring and synthetically designed peptides [4, 10, 21]. These insights inspired the development of the fragment-based approach for peptide representation. Our graph framework treats these fragments as a distinct class of nodes, with the inter-fragment bonds forming the graph edges. The formed fragment view implicitly captures the properties of non-canonical amino acids.

**Figure 1:**
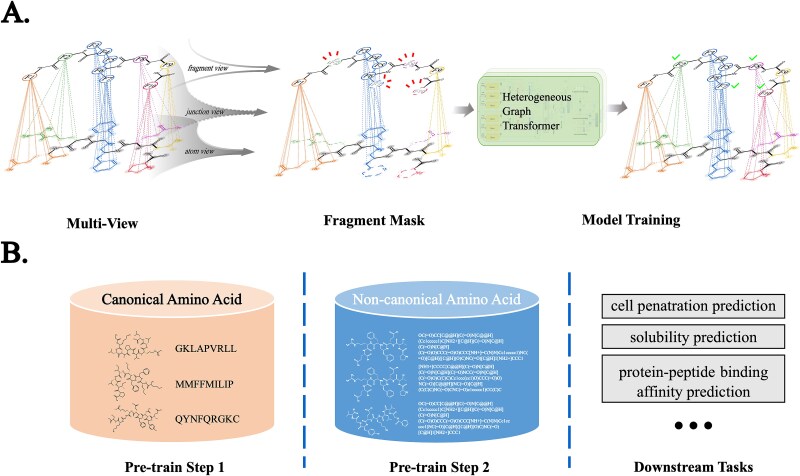
The overall workflow of the proposed PepLand framework. (A) PepLand uses a multi-view heterogeneous graph network to represent the molecular graph of peptides. Fragments of various granularities will be randomly masked for self-supervised pre-training. (B) Two-stage training approach. PepLand will be firstly trained on peptide sequences containing only canonical amino acids, and then further trained on peptide sequences containing non-canonical amino acids. After this, PepLand can be fine-tuned for downstream property prediction tasks.

As shown in Fig. 1A, the training phase employs the attribute masking strategy [22], wherein node/edge attributes like atom types are randomly masked, and the graph neural network (GNN) model is challenged to predict these masked attributes. This strategy is crucial for peptide representation learning due to the structural repetitions in peptides, such as recurring peptide bonds, which render the atom-level random masking unsuitable. For example, a simplistic prediction of atoms in amino bonds would be trivial due to their abundance in peptides. We have devised a specialized masking strategy that caters to the unique structure of peptides to address this issue.

It should be noted that the dataset size for non-canonical amino acids available in the literature is relatively small. Consequently, the training process is divided into two sub-phases, as demonstrated in Fig. 1B. The initial pre-training phase utilizes data comprising canonical amino acids, which lays the foundation for the model to discern peptide structural properties. The second phase incorporates peptides with non-canonical amino acids, which tine-tunes the model’s ability to recognize and process a broader spectrum of molecular structures. This dual-phase training ensures that our model is proficient in handling both canonical and non-canonical peptides through the enhanced capability in extracting meaningful features from a diverse range of peptides.

The detailed descriptions of the main modules are given in the section ‘Main Modules of PepLand’ in the Supplementary Materials.

## Results and discussion

### Assessing graph pooling techniques in PepLand

**Figure 2:**
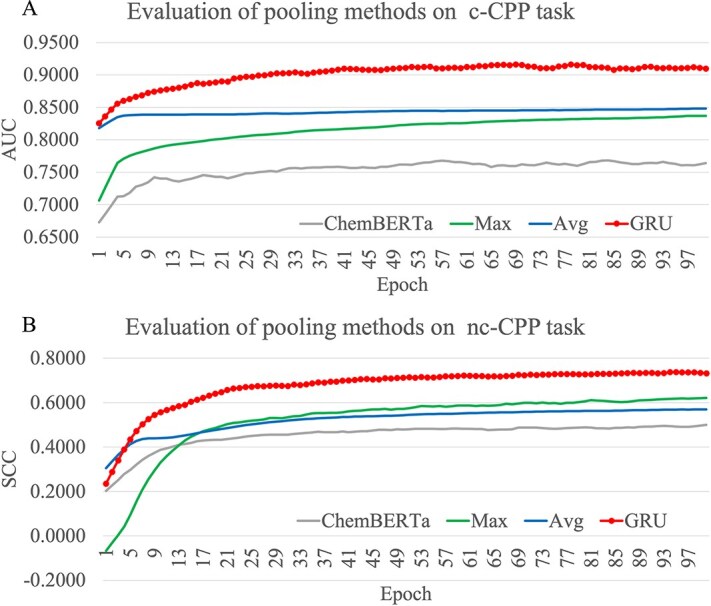
Impact of graph pooling method selection on downstream tasks. The figures illustrate the comparative effects of the ChemBERTa model, and its three variants with the graph pooling method replaced by max pooling (Max), average pooling (Avg), and gated recurrent unit pooling (GRU) across four prediction tasks, respectively. The horizontal axis gives the training epochs, and the vertical axis gives the values of the performance metrics. The evaluated prediction tasks are (A) c-CPP and (B) nc-CPP. Both prediction tasks use Spearman correlation coefficient (SCC) as the performance metrics.

This study designed a GNN-based pre-training methodology PepLand to generate peptide representations at both the atomic and fragment levels, rather than directly representing peptides at the amino acid level. In addition, numerous studies have demonstrated that graph pooling is crucial for acquiring a comprehensive graph-level representation of the entire graph [23, 24]. Therefore, in this section, we investigated the five prediction tasks using the PepLand-based peptide representations based on three graph pooling techniques, i.e. Max, Average (Avg), and gated recurrent unit (GRU). Figure 2A and B illustrates the results for the two prediction tasks c-CPP and nc-CPP, and Supplementary Fig. S1 shows the comparison on all the five prediction tasks. At the same time, for the purpose of comparison, we have also displayed the results of ChemBERTa-based peptide representation in the two Figures.

The experimental data in Fig. 2 indicates that the GRU pooling excels the other three methods on both prediction tasks c-CPP and nc-CPP at most of the training epochs. Except for the Avg pooling before the fifth epoch on the nc-CPP task, the GRU pooling outperforms all the three methods on both prediction tasks at all the training epochs. The GRU pooling achieves at least 18.67% improvements than the other three methods on the non-canonical task nc-CPP, while a minimum improvement of 8.00% is achieved by the GRU pooling on the c-CPP prediction task. The GRU pooling improves the best of the other three methods by at least 10.02% on the non-canonical task nc-Binding, and a smaller improvement 6.29% is achieved by the GRU pooling on the canonical task c-Binding, as shown in Supplementary Fig. S1. It is worth mentioning that regardless of the pooling method used, PepLand-based peptide representations consistently outperform ChemBERTa-based peptide representations. This indicates that the pooling method is not the only reason for the superior performance of PepLand.

The GRU pooling method is used in the following sections based on the experimental data.

### Benchmarking PepLand against existing models

Table 1 demonstrates the notable performance improvements achieved by our PepLand model in comparison to the existing studies, particularly in the prediction tasks involving non-canonical amino acids. PepLand achieves 0.628 in SCC for the prediction task nc-CPP, while the three existing models Uni-Mol [25], ChemBERTa2 [26], and MolCLR [27] only achieve the SCC values of 0.539, 0.503, and 0.461, respectively. A significant improvement 16.5% is achieved by PepLand against the second best model Uni-Mol. PepLand achieves the best SCC value 0.768 for the prediction task nc-Binding, while the second best model ChemBERTa2 only achieves 0.721 in SCC. The data clearly underscores the superior capability of PepLand in capturing the intricacies of non-canonical amino acids.

**Table 1:** Performances of the PepLand models and the existing studies on the five prediction tasks

	Spearman	Spearman		AUC	AUC	Spearman
Method	nc-CPP	nc-Binding	Method	c-CPP	c-Sol	c-Binding
	SCC	SCC		AUC	AUC	SCC
MolCLR	0.461	0.707	ESM2	**0.885**	0.725	0.452
ChemBERTa2	0.503	0.721	Uni-Mol	0.732	0.646	0.411
Uni-Mol	0.539	0.687	MolCLR	0.728	0.632	0.446
PepLand	**0.628**	**0.768**	ChemBERTa2	0.773	0.549	0.488
			PepLand	0.838	0.662	**0.503**
			Uni-Mol + ESM2	0.854	0.697	0.445
			MolCLR+ESM2	0.786	0.712	0.439
			ChemBERTa2 + ESM2	0.821	0.562	0.471
			PepLand+ESM2	**0.885**	**0.73**	0.454

The two prediction tasks c-CPP and c-sol are evaluated by the metric AUC, and the other three tasks are evaluated by SCC. Both AUC and SCC show a better prediction model with a larger value. The best value for each prediction task is highlighted in bold. The second-best results are underlined.

PepLand outperforms the other small molecule-based models, i.e. Uni-Mol, MolCLR, and ChemBERTa2, across the three prediction tasks involving canonical amino acids, i.e. c-CPP, c-Sol, and c-Binding. However, PepLand performs worse than the protein language model ESM2 [28] on the two prediction tasks c-CPP and c-Sol. This might be attributed to the extensive training of ESM2 on the UniRef50 dataset [29], encompassing ~45 million protein sequences through a vast parameter count of 15 billion. PepLand was trained on a dataset of only 8 million peptides and did not utilize the extensive evolutionary information encoded in ESM2. Nevertheless, ESM2 is not equipped to process non-canonical amino acids, a functionality inherent to our PepLand model.

To demonstrate that PepLand’s features provide additional value for tasks involving canonical peptides, we further explored the synergistic integration of PepLand and ESM2, denoted as PepLand+ESM2. When integrating PepLand with ESM2, the combined model achieves the best AUC of 0.885 on the c-CPP task and 0.73 on the c-Sol task, while showing a slight decrease in SCC for c-Binding (SCC = 0.454) compared to standalone PepLand. Notably, other combinations with ESM2, such as Uni-Mol + ESM2, MolCLR+ESM2, and ChemBERTa2 + ESM2, do not yield as significant improvements. For example, Uni-Mol + ESM2 achieves an AUC of 0.854 on c-CPP and 0.697 on c-Sol, while ChemBERTa2 + ESM2 achieves an AUC of 0.821 on c-CPP but performs poorly on c-Sol with an AUC of 0.562. These experiments confirm that PepLand’s features uniquely complement ESM2, resulting in more consistent performance improvements compared to other models.

Overall, these results highlight the effectiveness of PepLand, particularly in its handling of non-canonical peptides. The PepLand-based representation also demonstrates its potential as a valuable contribution for the canonical peptide-based prediction tasks.

### Importance of fragmentation granularity

**Figure 3:**
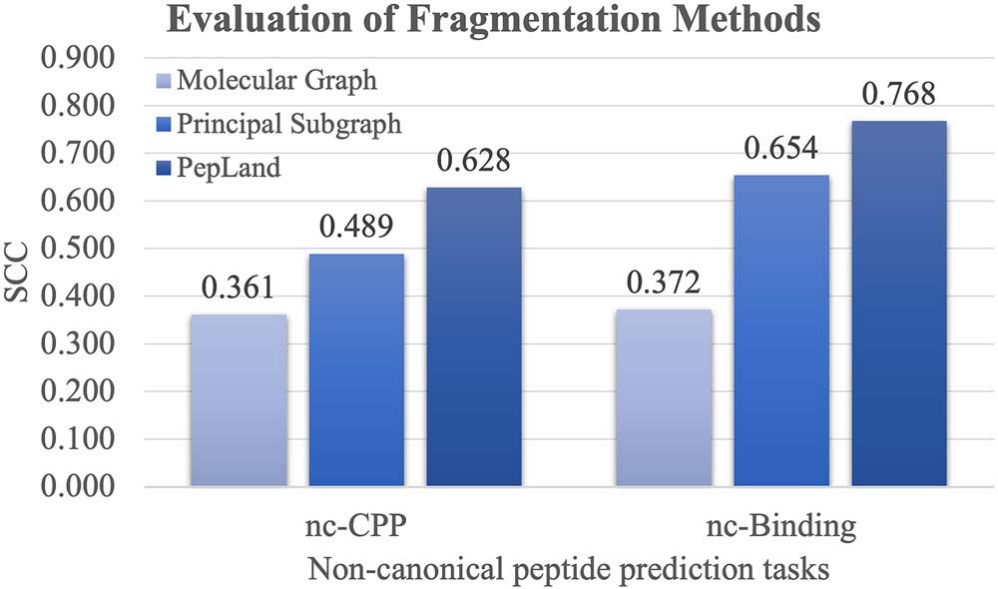
Effects of different fragmentation methods on the two non-canonical peptide-based prediction tasks. The two tasks are nc-CPP and nc-binding, which are listed in the horizontal axis. The vertical axis gives the performance metric SCC.

Molecular fragmentation plays a key role in the process of developing drugs using artificial intelligence [30]. PepLand uses a fragmentation granularity that lies between atomic and complete amino acids. Moreover, PepLand’s fragmentation operator is not only data-driven, but also integrated with domain knowledge. This section aims to compare PepLand’s superiority with other granular and purely data-driven fragmentation methods. We employ two additional fragmentation methods as benchmarks against our PepLand model. The first method is termed ‘Molecular Graph’, representing a baseline atomic granularity approach. The second method ‘Principal Subgraph’ [15] is a data-driven fragmentation approach designed to autonomously identify frequent principal subgraphs within the dataset. Figure 3 evaluates three fragmentation methods for the two tasks nc-CPP and nc-Binding.

To maintain consistency and fairness across comparisons, we preserve the identical training and testing subsets across all the fragmentation methods, and only change the molecular fragmentation methods. The scenario of Molecular Graph has the singular focus on atom representation, and operates with a single-view graph structure.

The comparative analysis reveals that the Molecular Graph approach yields the least effective outcomes within its atomic granularity. This inferior performance is likely attributable to the complexity and larger structures of peptide molecules, which could lead to significant information loss during the message passing and pooling stages in the GNN framework. The Principal Subgraph method employs a sophisticated data-driven fragmentation and does not integrate explicit domain knowledge. Figure 3 shows that the Principal Subgraph method achieves the substantial improvements of 0.128 and 0.282 in the SCC values over the baseline Molecular Graph method. This supports the necessity of appropriate molecular fragmentation the non-canonical peptide prediction tasks.

PepLand further enhances the Principal Subgraph method through the employment of tailored fragmentation and domain knowledge (Fig. 3). The SCC values of the two prediction tasks nc-CPP and nc-Binding are increased to 0.628 and 0.768, respectively. This suggests that an optimal balance between fragmentation detail and domain knowledge is key to effectively capturing the subtle patterns within peptide structures, especially when dealing with the varied and complex nature of non-canonical peptides.

### Evaluating the efficacy of fragmentation operators

**Figure 4:**
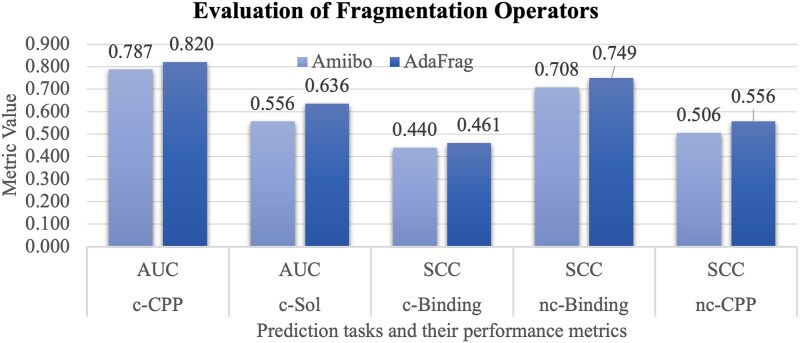
Comparative analysis of the two fragmentation operators on downstream tasks. The horizontal axis gives the five prediction tasks and their performance metrics. The vertical axis gives the metric value. The two fragmentation operators Amiibo and AdaFrag are evaluated.

This section assesses the contributions of the two fragmentation operators to the five downstream tasks, as shown in Fig. 4. The PepLand framework without further fine tuning is used for the prediction tasks.

The Amiibo operator has already outperformed the Molecular Graph and the Principal Subgraph models on the two prediction tasks on non-canonical peptides. The Amiibo operator achieves the SCC values of 0.506 and 0.708 for the downstream tasks nc-CPP and nc-Binding. The Molecular Graph and Principal Subgraph models only achieve the SCC values of 0.361 and 0.489 for the nc-CPP tasks, and the SCC values of 0.372 and 0.654 for the nc-Binding tasks.

Figure 4 provides compelling evidence that the integration of the AdaFrag operator significantly enhances the PepLand framework’s performance. This is particularly evident in the Spearman correlation coefficient (SCC) values, which reach 0.556 and 0.749 for the two canonical peptide-based tasks nc-CPP and nc-Binding, respectively. The underlying strategies of the Amiibo and AdaFrag operators are fundamentally different yet complementary. While the Amiibo operator focuses on preserving the structural integrity of amino bonds, thus encapsulating key biochemical knowledge, AdaFrag adopts a data-driven approach using the Breaking of Retrosynthetically Interesting Chemical Substructures (BRICS) algorithm [31] to identify and leverage frequently occurring fragments. The results highlight the crucial role of blending biological knowledge with patterns inherent in the data. Such a combination not only respects the fundamental biochemical properties of peptides but also adapts to the empirical regularities discovered in large-scale peptide datasets. This synergy between domain-specific knowledge and data-driven insights is pivotal in advancing the accuracy and relevance of peptide representation models like PepLand.

### Assessing the effects of different masking strategies

**Figure 5:**
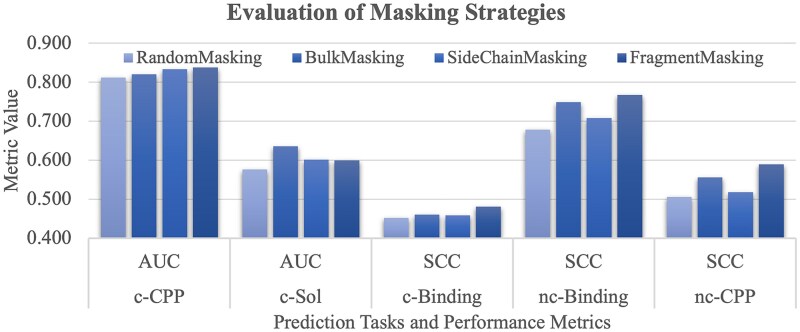
Assessing the effects of different masking strategies on the downstream tasks. The horizontal axis gives the five prediction tasks and their performance metrics. The vertical axis gives the metric value. This figure evaluates four masking strategies, i.e. RandomMasking, BulkMasking, SideChainMasking, and FragmentMasking.

We extend our investigation to examine how performance of downstream tasks is affected by the four masking strategies, i.e. RandomMasking, BulkMasking, SideChainMasking, and FragmentMasking (Fig. 5).

RandomMasking performs the worst across all the five prediction tasks. This indicates that the inherent connections among molecular atoms are not captured by the random masking strategy. The BulkMasking strategy achieves the best performance 0.636 in AUC for the c-Sol task and the second best performance on the two non-canonical peptide-based tasks nc-CPP and nc-Binding. Figure 5 shows that FragmentMasking outperforms the other three masking strategies across four tasks except for c-Sol.

It is anticipated that FragmentMasking achieves the best performance 0.589 and 0.767 in SCC for the two non-canonical peptide-based tasks nc-CPP and nc-Binding.

### Evaluating the two-step pre-training

**Figure 6:**
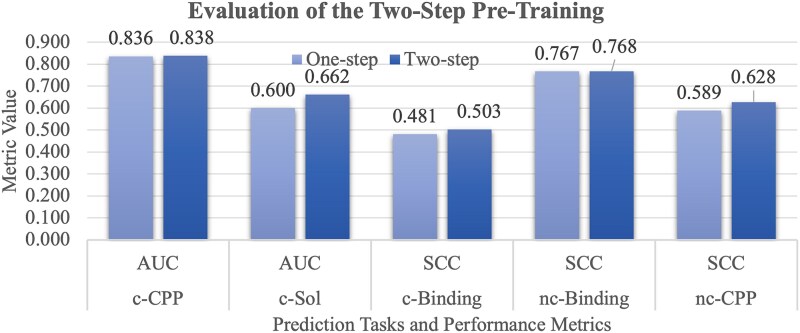
Evaluation of the two-step pre-training. The horizontal axis gives the five prediction tasks and their performance metrics. The vertical axis gives the metric value. This figure evaluates two scenarios, i.e. one-step only utilizes the first pre-training step, and two-step fully utilizes the two pre-training steps in this study.

This study employs a two-step pre-training procedure, whose necessity is evaluated in Fig. 6. Our focus is to determine the effectiveness of the two pre-training steps in extracting the rich peptide information, particularly for non-canonical peptides.

The data shown in Fig. 6 indicate that the two-step pre-training strategy outperforms the single-step approach across all the five benchmark tasks. The largest improvement 0.062 in AUC is achieved on the prediction task c-Sol, and the second best improvement 0.039 in SCC is achieved on the nc-CPP task.

### Assessing the effects of the multi-view feature fusion strategies

This section investigates the role that multi-view feature fusion plays in downstream task performance. Our model integrates three distinct feature views, and we experiment with various combinations of these multi-view features to ascertain the most effective fusion strategy. After evaluating performance across five downstream tasks, we find that the integration of both atom embeddings and fragment embeddings with the junction-view (AJ&FJ) emerges as the most effective fusion approach.

**Figure 7:**
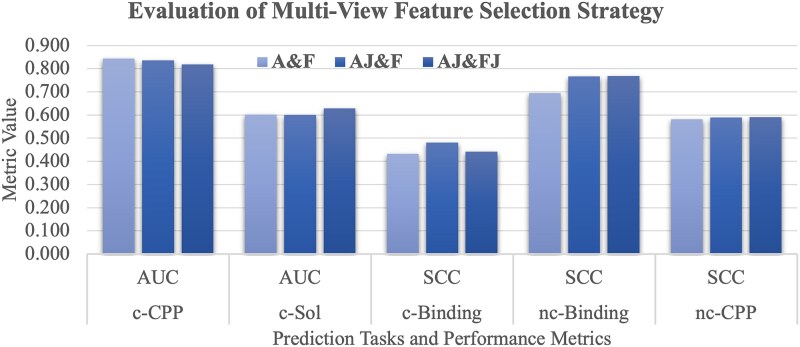
Evaluation of the multi-view feature fusion strategies. The horizontal axis gives the five prediction tasks and their performance metrics. The vertical axis gives the metric value. This figure evaluates three multi-view fusion strategies, i.e. A&F, AJ&F, and AJ&FJ, where A, J, and F refer to the atom-, junction-, and fragment-view. AJ and FJ represent the atom embeddings and fragment embeddings with the junction-view features, respectively.

Figure 7 shows that the AJ&FJ fusion strategy achieves the best performance on three prediction tasks, while the A&F and AJ&F strategies reach the best performance on only one task. The AJ&FJ fusion strategy outperforms the other strategies on both non-canonical peptide-based tasks, nc-Binding (SCC = 0.769) and nc-CPP (SCC = 0.591).

This exploration experiment highlights the significance of strategic view fusion strategies in our multi-view graph model. The carefully-selected fusion strategy of the three feature views (atom, junction, and fragment) can harness the strengths of each view to enhance the overall representation power of the model. The default fusion strategy AJ&FJ in our PepLand model performs particularly well in complex tasks involving non-canonical peptides, where the intricate structural and functional patterns of peptides are critical.

### Real-world generalization and application consistency of PepLand

To further assess the practical utility and generalizability of PepLand beyond controlled benchmark settings, we conducted additional experiments focusing on real-world peptide discovery tasks. While PepLand achieves performance comparable to state-of-the-art models such as ESM2 on canonical peptide property benchmarks (e.g. c-CPP and c-Sol), the differences between models on these tasks are relatively small. This prompted us to explore new datasets and scenarios that better reflect the complexities of real-world peptide applications.

We identified three newly published peptide bioactivity classification datasets introduced in the UniDL4BioPep framework [32], covering ACE inhibitory, bitter, and umami peptides. These datasets were designed to simulate realistic peptide screening tasks and feature peptide lengths ranging from 0 to 50 residues—consistent with common therapeutic and food-derived peptides. We evaluated PepLand in combination with ESM2 (PepLand+ESM2) using a linear probe setting to simulate low-data transfer learning conditions. Across all three datasets, PepLand+ESM2 consistently outperform ESM2 alone, as shown in Table 2.

**Table 2:** Performance comparison of PepLand on real-world peptide bioactivity classification datasets.

Dataset	Model	ACC	ROC-AUC
ACE inhibitory	ESM2	0.812	0.843
	Pepland + ESM2	**0.823**	**0.865**
Bitter	ESM2	0.886	0.935
	Pepland + ESM2	**0.895**	**0.947**
Umami	ESM2	0.823	0.878
	Pepland + ESM2	**0.846**	**0.894**

Bold values indicate the best performance among all methods.

These results demonstrate that PepLand contributes transferable representations that improve generalization across multiple real-world peptide tasks, including functional property prediction beyond simple physicochemical attributes.

In addition to these sequence-based classification tasks, we also evaluate the performance of PepLand on binding affinity prediction for linear peptides using data from the SKEMPI 2.0 database [33], as detailed in Case Study 3 in the Supplementary Materials. This study includes 10 sets of protein–peptide mutational datasets with varying peptide lengths and sample sizes. Our model achieve strong performance on mid-size datasets, e.g.: 3EQS (SCC = 0.927), 3EQY (SCC = 0.916) and 1F47 (SCC = 0.516). Perfect SCCs on very small sets (e.g. 3LNZ, 1GL0), which are likely overfitted due to small sample sizes. These findings suggest that while PepLand generalizes well to external peptide–protein affinity tasks, its performance varies with data scale and peptide diversity—pointing to the need for further extension of training data to improve robustness.

## Conclusion

This study introduces PepLand, a pre-trained model capable of extracting features of both canonical and non-canonical peptides. Unlike current protein language models, this model can process both canonical and non-canonical amino acids simultaneously. To our knowledge, this is the first pre-trained model specifically designed for extracting representations of non-canonical peptides. The study demonstrates the importance of molecular fragmentation granularity and develops the fragmentation method AdaFrag, which captures the characteristics of non-canonical peptides more effectively. Compared to atomic-level fragmentation and purely data-driven fragmentation, AdaFrag significantly improves the performance of the non-canonical peptide-based prediction tasks nc-CPP and nc-Binding, with increases of 28.4% and 17.4% respectively, relative to the second-best Principal Subgraph approach. PepLand employs a multi-view heterogeneous graph as the main architecture and customizes fragment masking and two-step training strategies based on the characteristics of non-canonical peptides. The comprehensive ablation experiments validate the efficacy of each component of PepLand. For more detailed information, please refer to the Supplementary Figs S2-S5.

PepLand shows a particular proficiency in handling non-canonical peptides that incorporate non-canonical amino acids, a domain where PepLand unequivocally excels. In the prediction of cell penetration ability and binding affinity containing non-canonical amino acids, PepLand achieved 16.5% and 6.5% higher than the second-best model, respectively. Another interesting point is, despite its training set being much smaller than ESM2, PepLand outperforms ESM in some peptide property prediction tasks involving only canonical amino acids, such as reaching an SCC of 0.503 in the c-CPP task, while ESM2 only achieved 0.452. More importantly, the representations learned by PepLand seem to complement the co-evolutionary information learned by ESM2, as the combination of their representations achieves the best performance on the c-CPP and c-Sol tasks.

A number of limitations remain to be resolved in future studies. First, while AdaFrag’s tokenizer learns molecular fragments based on the training data, it can encounter out-of-vocabulary issues for fragments not observed during training. We are aware of this limitation and will work toward improving the tokenizer’s coverage in upcoming versions. For larger fragments, AdaFrag leverages the BRICS algorithm to further split fragments into smaller subunits, as demonstrated in Supplementary Fig. S8. Second, the diversity of our training data might not fully capture the broad scope of synthetic peptides, particularly those containing rare or recently identified non-canonical amino acids. Although our fragmentation method is flexible and can handle certain atypical amino acids beyond existing datasets, it does not entirely overcome this challenge. Finally, our training dataset, consisting of <10000 synthetic peptides (mostly transmembrane peptides), underscores the need for more extensive data collection. We believe that integrating additional datasets in the future will enhance PepLand’s performance and broaden its range of applications.

Additionally, the computational efficiency of PepLand represents another major limitation. As a GNN-based model, its tokenizer converts molecules into heterogeneous graphs. This process, while integral to the model’s design, represents the primary bottleneck in performance, particularly for new data, as it involves graph construction and feature extraction. Compared to models like ESM2, which directly take amino acid sequences as input, PepLand’s average inference speed is 7.38 times slower on the c_CPP dataset. Furthermore, the scalability of GNNs is inherently constrained by their message-passing mechanism, as documented in the literature [34, 35]. These factors highlight the need for future work to optimize the tokenization process and address scalability challenges.

The first two case studies in the supplementary materials demonstrate particularly promising outcomes in the predictions of cyclic peptide binding affinity and peptide synthesizability, highlighting the potential of PepLand for a broad spectrum of applications. Additionally, a third case study using linear peptide data further showcases the model’s practical utility. However, we acknowledge that the current evaluation is based on a limited number of case studies and datasets, which only cover a subset of realistic peptide discovery scenarios. In particular, the scale and diversity of real-world evaluation datasets remain limited, which constrains our ability to draw broader conclusions about generalization. Looking forward, we emphasize the importance of establishing broader and more diverse benchmarking settings to better reflect the wide range of practical peptide discovery applications. Despite current limitations, the development and successful validation of PepLand provide a strong foundation for integrating machine learning into peptide research, particularly for studies involving non-canonical amino acids. We anticipate that PepLand will inspire the creation of more advanced models and applications, driving the exciting field of peptide representation into new frontiers.

**Key Points**

- Introduce a novel GNN framework PepLand for representing peptides of both canonical and non-canonical amino acids.
- PepLand integrates two molecular fragmentation operators to discern the optimally granular elements within peptide structures.
- A two-step training strategy is employed to ensure learning efficiency, even with the relatively limited data on non-canonical amino acids.
- Comprehensive evaluations and case studies demonstrate the effectiveness of the pre-trained PepLand model.
- The source code and datasets are publicly available to facilitate future studies on peptide representations and peptide design.

## Supplementary Material

PepLand_r2_supp_bbaf367

## Data Availability

We have made the entire source code available at http://www.healthinformaticslab.org/supp/resources.php or https://github.com/zhangruochi/PepLand.
