# Supplementary material for: PepLand: a large-scale pre-trained peptide representation model for a comprehensive landscape of both canonical and non-canonical amino acids
Source: Brief Bioinform. 2025 Aug 1;26(4):bbaf367. doi: 10.1093/bib/bbaf367 (PMC12315545; doi:10.1093/bib/bbaf367)
Supplement: PepLand_r2_supp_bbaf367 [file pepland_r2_supp_bbaf367.docx]

# PepLand: a large-scale pre-trained peptide representation model for a comprehensive landscape of both canonical and non-canonical amino acids

Ruochi Zhang^1,2,3^, Haoran Wu^3^, Chang Liu^4^, Qian Yang^1,5^, Yuting Xiu^3^, Kewei Li^1,5^, Ningning Chen^3^, Yu Wang^3^, Yan Wang^1,2,5^, Xin Gao^6,7,*^, Fengfeng Zhou^1,5,*^.

1 Key Laboratory of Symbolic Computation and Knowledge Engineering of Ministry of Education, Jilin University, Changchun, Jilin, China, 130012.

2 School of Artificial Intelligence, Jilin University, Changchun, China,130012.

3 Syneron Technology, Guangzhou, China, 510700.

4 Beijing Life Science Academy, Beijing 102209, China.

5 College of Computer Science and Technology, Jilin University, Changchun, Jilin, China, 130012.

6 Computational Bioscience Research Center, King Abdullah University of Science and Technology (KAUST), Thuwal, Saudi Arabia, 23955.

7 Computer Science Program, Computer, Electrical and Mathematical Sciences and Engineering Division, King Abdullah University of Science and Technology (KAUST), Thuwal, Saudi Arabia, 23955.

# Correspondence may be addressed to Fengfeng Zhou: FengfengZhou@gmail.com or ffzhou@jlu.edu.cn . Lab web site: http://www.healthinformaticslab.org/. Phone: +86-431-8516-6024. Fax: +86-431-8516-6024. Correspondence may also be addressed to Xin Gao: xin.gao@kaust.edu.sa.

# Supplementary Figure S1

Impact of graph pooling method selection on downstream tasks. The figures illustrate the comparative effects of the ChemBERTa model, and its three variants with the graph pooling method replaced by max pooling (Max), average pooling (Avg), and gated recurrent unit pooling (GRU) across four prediction tasks, respectively. The horizontal axis gives the training epochs, and the vertical axis gives the values of the performance metrics. The evaluated prediction tasks are (a) c-CPP, (b) nc-CPP, (c) c-Sol, (d) c-Binding, and (e) nc-Binding. The c-CPP and c-Sol prediction tasks use AUC as the performance metric, and the other prediction tasks use Spearman correlation coefficient (SCC) as the performance metrics.

(a)

(b)

(c)

(d)

(e)

# Supplementary Figure S2

The figure demonstrates the impact of the fragment splitting strategy on downstream tasks.

# Supplementary Figure S3

The figure illustrates the influence of the masking method in pre-training models on downstream tasks.

# Supplementary Figure S4

The figure presents the impact of the two-stage pre-training on downstream tasks.

# Supplementary Figure S5

The figure illustrates the selection of multi-view features in the model, where A represents atom-level features, J represents junction-level features, and F represents fragment-level features.

# Main Modules of PepLand

## **Heterogeneous Graph-based Peptide Representation**


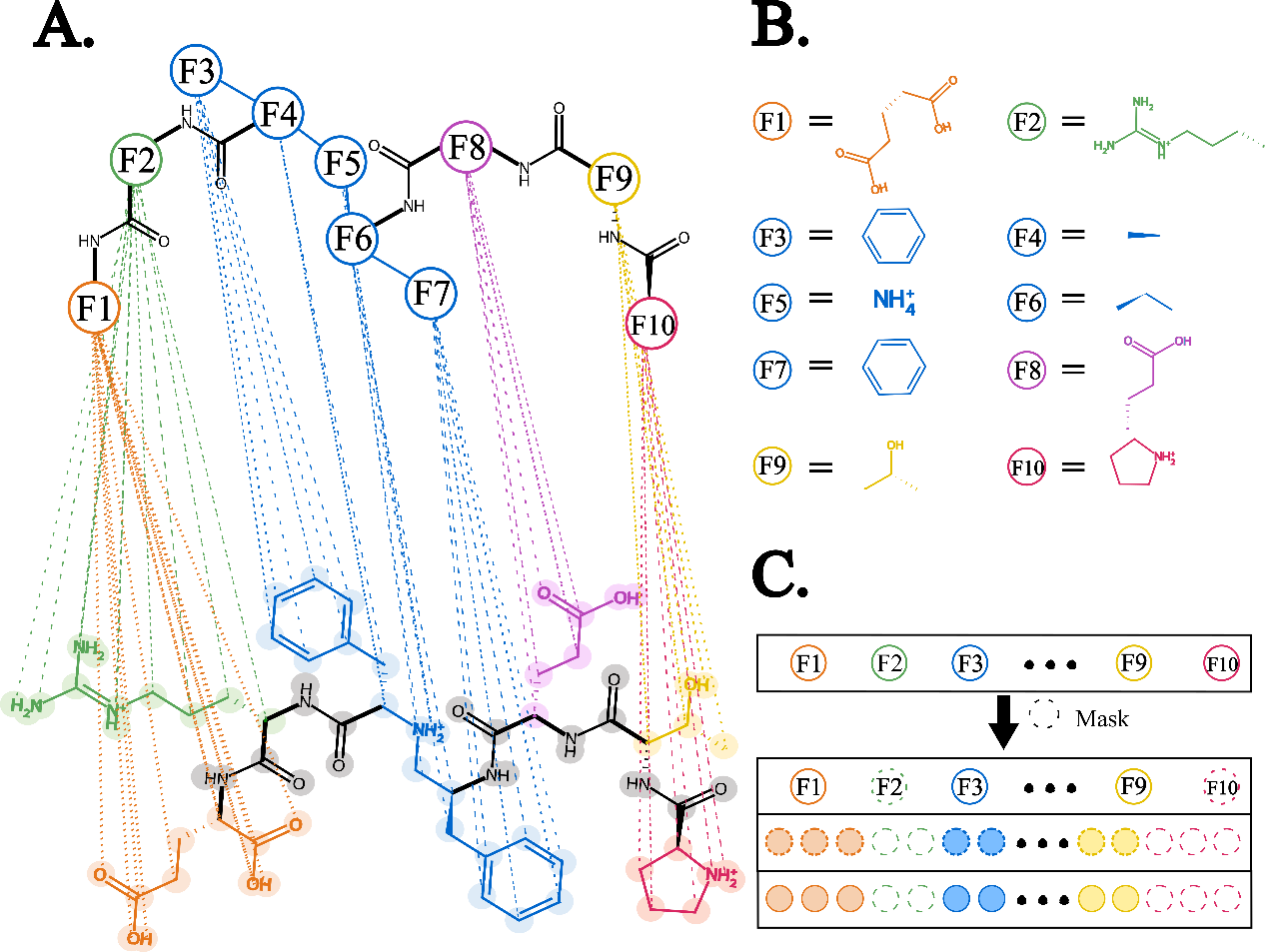


Supplementary Figure S6. The multi-view feature representation framework of PepLand. (A) A peptide molecule can have multiple representation views. The top F1-F10 in the figure represent the fragment view, and the bottom part represents the atom-level view. Both atoms and fragments will facilitate the learning of the junction representation view. Homogeneous edges are formed within atoms and fragments. Heterogeneous edges are formed between atoms and fragments, as each fragment in the figure is connected to a specific subgraph structure below. (B) Molecular graph structures of F1-F10. They are connected by amino bonds. (C) Each representation view will be randomly masked for self-supervised learning.

Supplementary Figure S6 visually depicts the proposed heterogeneous graph neural network PepLand based on the effective integration of atoms and fragments for peptide representation. Heterogeneous graph structure combining atoms and fragments has been successfully explored for molecular representation learning [1]. Both atoms and fragments constituting a peptide are represented as nodes in PepLand. The intricate network of relationships (or edges) among these components include chemical bonds linking atoms, connections between fragments, and junctions between atoms and fragments. These junction edges ensure that a fragment is connected exclusively to its constituent atoms. The design aims to effectively transmit features across different granularities through the message passing mechanism, and to enhance the model’s capacity to capture critical patterns on both the atom and fragment levels of peptides.

Supplementary Figure S7. The message passing mechanism of the multi-view graph representation un-supervised learning in the PepLand framework.

We employ the similar message passing mechanism proposed in the Pharmacophoric-constrained Heterogeneous Graph Transformer (PharmHGT) [2], as shown in Supplementary Figure S7. This approach is distinct from the classical Heterogeneous Graph Transformer (HGT) model [3] in the integration of edge features into the GNN’s message passing mechanism. The node features are denoted as $X_{V_{i}}$, the edge features are represented as $X_{e_{ij}}$, the hidden states of edges are denoted as $H\left( X_{e_{ij}} \right)$, and the hidden states of nodes are denoted as $H\left( X_{V_{i}} \right)$. The messaging steps for information propagation are outlined as follows:

$M_{V}^{1}(X_{vi}) = \sum_{\theta_{\mathcal{N(}V_{i})}} H\left( X_{\theta_{\mathcal{N}}\left( V_{i} \right)} \right), t=1$ (1)

$M_{E}^{1} \left( X_{e_{ij}} \right)=H\left( X_{v_{i}} \right), t=1$ (2)

$M_{V}^{t}(X_{vi}) = \sum_{\theta_{\mathcal{N}}(V_{i})} Attention\left( H^{t-1}\left( X_{v_{i}} \right) \right)W_{v_{i}}^{Q}, t>1$ (3)

$M^{t-1}\left( X_{\theta_{\mathcal{N}}\left( V_{i} \right)} \right)W_{m_{i}}^{K}, H^{t-1}\left( X_{v_{i}} \right)W_{v_{i}}^{V}, t>1$ (4)

$M_{E}^{t}\left( X_{e_{ij}} \right)= Linear\left( M_{E}^{1} \left( X_{e_{ij}} \right) \right)+ H^{t}\left( X_{vi} \right)-H^{t-1}\left( X_{e_{ij}} \right), t>1$ (5)

Here the $\theta_{\mathcal{N(}V_{i})}$ is a function identifying edges directed toward node *v_i_* and the relevant messages are $M_{E}^{t}\left( X_{e_{ij}} \right)$ and $M_{V}^{t}(X_{vi})$. In order to mitigate the potential vanishing gradient issue, our model integrates a basic residual block during the multi-view message passing process:

$\left\{ \begin{aligned} H^{t}\left( X_{vi} \right)= H^{t-1}\left( X_{vi} \right)+ M_{V}^{t}(X_{vi}) \\ H^{t}\left( X_{e_{vi}} \right)= H^{t-1}\left( X_{e_{vi}} \right)+ M_{E}^{t}(X_{e_{vi}}) \end{aligned} \right.$ (6)

## **Fragmentation Operators**

The previous sections have elucidated the significant role of integrating fragment information with atom-based molecular graphs to augment the representational power of models, particularly for peptides containing non-canonical amino acids. However, the definition of a fragment within this context is not straightforward, as they can vary in sizes and types. The fragmentation choice is pivotal in determining the properties of the resultant polypeptides [4]. This study establishes two key objectives for designing a fragmentation method. First, the generated fragments should enhance and supplement the existing features between the atomic and amino acid levels through the incorporation of domain-specific knowledge. For instance, the six carbons in a benzene ring are often regarded as a hydrophobic entity [5], which could be advantageous in graph-based pre-training and various downstream property prediction tasks. Second, the fragment library should have an optimal size in terms of vocabulary. The frequency distribution of fragments in the data should avoid a long-tail pattern, as such distributions could not evenly distribute the representation burdens of different fragments and could potentially complicate the model’s learning process.


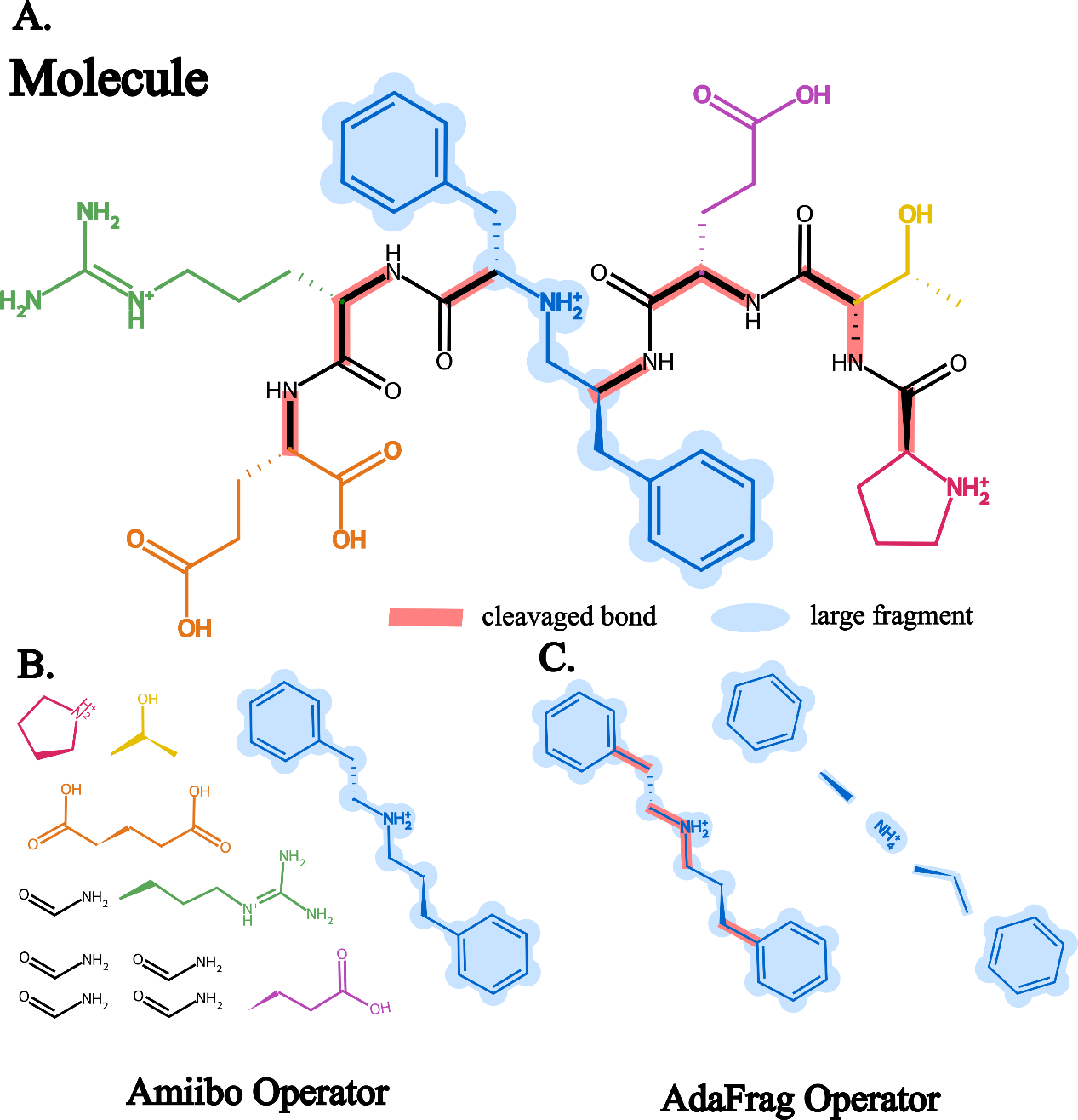


Supplementary Figure S8. Illustrations of the Amiibo and AdaFrag fragmentation operators. Amiibo operator breaks the molecules while preserving amino bonds, and continues to further fragment large side chains using the BRICS algorithm [6]. (A) An example molecular graph of a peptide containing non-canonical amino acids, with each side chain highlighted in different colors. The Amiibo operator breaks all the cleavage bonds, preserving all amino bonds, and cut the molecule into multiple fragments. (B) Output of the Amiibo operator. It can be observed that, in addition to some peptide bonds and common side chains, there is a larger fragment covered by blue color. (C) The AdaFrag operator will further use the BRICS algorithm to break this large fragment.

To meet these objectives, we developed two fragmentation operators: Amiibo and AdaFrag. These operators provide a systematic approach to obtaining fragments with appropriate granularity, as illustrated in Supplementary Figure S8.

1. **Amiibo (Amino Bond Preservation):**
   - The first step involves identifying the molecular graph of a peptide, where each side chain is highlighted in a distinct color to differentiate them (Figure S8A).
   - The Amiibo operator then systematically breaks all cleavage bonds, preserving amino bonds as the structural core of the fragments. This results in multiple fragments that retain the integrity of amino bonds while separating functional groups or side chains from the peptide backbone (Figure S8B).
   - For example, in Figure S8B, the blue-highlighted fragment represents a large group retained after this step due to its complex structure, which is common for non-canonical amino acids.
2. **AdaFrag (Adaptive Fragmentation):**
   - For larger fragments, such as the blue-highlighted group from Figure S8B, the AdaFrag operator applies the BRICS algorithm to further break them into smaller substructures. This process leverages predefined rules to fragment complex structures into manageable sizes for better representations of non-canonical side chains (Figure S8C).
   - By further refining large fragments, the AdaFrag operator facilitates more uniform fragment distributions, avoiding long-tail patterns in the fragment library.

This step-by-step process ensures that the fragmentation method not only preserves critical structural motifs like amino bonds but also creates a fragment library optimized for representation learning. The combination of Amiibo and AdaFrag balances the trade-off between structural integrity and granularity, addressing the challenges of representing diverse peptide structures while enhancing model performance in downstream tasks.

Supplementary Table S1. Statistical data of the fragment library derived from two fragmentation operators. The first and second columns give the fragmentation operator and the numbers of fragments generated by the corresponding fragmentation operators. The third column “Ratio of Freq<=5” gives the percentage of fragments detected in at most 5 peptides in the dataset. The last column “Recovery Rate” is the percentage of peptides that can be reconstructed by the fragments in that line.

| Fragmentation | Size | Ratio of Freq<=5 | Recovery Rate |
| --- | --- | --- | --- |
| Amiibo | 410 | 37.10% | 88.60% |
| AdaFrag | 258 | 6.10% | 97.52% |

Supplementary Table S1 presents comprehensive statistical data for the fragment libraries derived from these two fragmentation operators, and offers insights into their respective efficiencies and applications.

The two fragmentation operators generate 410 (Amiibo) and 258 (AdaFrag) entries, respectively. However, over 37% of these Amiibo-generated fragments appear no more than five times. This reflects the coarse granularity of the Amiibo fragmentation operator. In contrast, only about 6% of the 258 AdaFrag-generated fragments occurring no more than five times. Fragments appearing two or fewer times are excluded from the libraries.

An essential metric in evaluating these fragmentation operators is the “recovery rate”, defined as the ability to reconstruct the original molecule using the fragments generated by a specific operator. The Amiibo operator demonstrates a lower recovery rate of 88.60%, primarily due to that its long-tailed frequency distribution leads to the exclusion of many infrequently occurring fragments. The AdaFrag operator substantially improves the recovery rate to 97.52%. For fragments not found within the fragment library, they are uniformly represented as OOV (out-of-vocab) fragments. This approach is a common practice in the field of Natural Language Processing (NLP) [7, 8]. It allows for the handling of unknown or rare fragments by providing a generic representation, thereby maintaining the integrity of the data analysis while accommodating the limitations inherent in the dataset's diversity. This method ensures that our system can still process and analyze molecular structures even when encountering fragments that have not been previously catalogued, enhancing the flexibility and adaptability of the model to new or rare molecular structures.

## **Masking Strategies**

Our study devises several masking strategies for the self-supervised training process inspired by the insights from [9]. These masking strategies are tailored for the unique context of peptide representation, and resemble the masked language modeling (MLM) strategy in natural language processing [10]. The MLM strategy randomly masks some tokens from the input text and the model is trained to predict these masked tokens based on the remaining unmasked tokens in the context.

However, the random masking strategy used in MLM is not entirely suitable for learning peptide representations, given the distinctive repetitive unites and specific sub-structures inherent in polypeptides. For example, amino acids are constituted by an amino group, a carboxyl group, and a side chain. The random masking strategy might mask parts of the amino or carboxyl groups, which are trivial to predict based on the other contextual atoms in the same amino or carboxyl groups. Therefore, we have designed the following specialized masking strategies and evaluated their respective performances:

1. RandomMasking: This method involves randomly masking individual atoms within the molecular graph. The challenge posed to the model in predicting the masked atom identities helps in learning robust peptide representations.

2. BulkMasking: This strategy masks all atoms that make up a single amino acid, and limits the availability of neighboring atom information to escalate the challenge in prediction. It mirrors the masking techniques used in the training of protein language models, with the critical difference being that it encompasses both canonical and non-canonical amino acids.

3. SideChainMasking: The peptide bonds’ atoms are a dominant feature in protein sequences, and the two strategies RandomMasking and BulkMasking often end up targeting these atoms. In order to further challenge the training process, the SideChainMasking strategy selectively masks atoms in the side chains while keeping the peptide bonds’ atoms unmasked. This strategy increases the complexity of the prediction task by focusing on the more variable elements of the peptide structure.

4. FragmentMasking: This strategy masks entire fragments within the peptide molecule. This compels the model to explore deeper into understanding of relationships and properties associated with the masked fragments.

Each of these masking strategies brings a unique dimension to the training process, and addresses different aspects of complexity and specificity in peptide representation. Their detailed evaluations offer valuable insights into the most effective methods for enhancing the predictive capabilities of our model.

## **Two-step Training Procedure**

This study implements a two-step training procedure to enhance the representation learning of peptides, especially those containing non-canonical amino acids. The initial step involves the model being trained exclusively on a dataset composed of canonical amino acids. This dataset consists of approximately 8 million sequences carefully curated from the UniProt database [11]. We classified protein sequences containing fewer than 50 amino acids as candidate peptide sequences for the training purpose. This step assimilates evolutionary information from a diverse pool of candidate peptide sequences.

However, the relatively limited and valuable presence of peptides with non-canonical amino acids necessitates a second step of training. This step focuses specifically on the unique characteristics of non-canonical amino acids

This two-step training procedure ensures that the model not only gains a comprehensive understanding of the broader peptide landscape but also develops specialized proficiency in recognizing and interpreting the subtle patterns in non-canonical peptides.

## **Linear Probes**

To assess the quality of the features derived from different pre-trained peptide models, we employed the linear probe technology [12]. This method employs the pre-trained model as a feature extractor to produce the feature representations $\mathcal{F}_{x}$ for a given set of labeled examples (*x*, *y*). A linear classifier or regressor is then trained on these features $\left( \mathcal{F}_{x}, y \right)$. This method assumes that good features should achieve a satisfying linear separation of classes or a linear relationship in regression in downstream tasks, and it allows the evaluation of feature quality independent of model architecture. This is critical that differences in predictive performance may arise from fine-tuned architectural advantages rather than superior pre-training.

# Evaluating Datasets

We conduct a rigorous evaluation of our pre-trained PepLand model through the curated array of datasets, spanning the prediction tasks of cell penetration ability, solubility, and protein-peptide binding affinity. Both canonical and non-canonical peptides are covered. We collected and curated these evaluation datasets from various databases and public literatures. To facilitate further research in the field of peptides, we have made these datasets publicly available together with our source code.

**Canonical Cell Penetrating Peptide (c-CPP)** Dataset: To create a dataset for evaluating the cell penetration ability of canonical peptide sequences, we select 1,162 transmembrane peptides from 22 cell-penetrating peptide databases [13, 14], excluding any sequences with non-canonical amino acids. For the construction of the negative set, we retrieve all protein sequences from UniProt and PeptideAtlas [15], applying a filter with a length threshold of 50. To avoid data duplication and ensure the integrity of our dataset, we perform deduplication using CD-HIT [16] at an 80% similarity threshold, both for individual datasets and when combining positive and negative samples. This process yielded a vast pool of 16,689,857 sequences. Finally, to balance our dataset, we sampled an equivalent number of negative samples based on the mean and variance of the positive samples' lengths, resulting in a well-balanced transmembrane peptide validation dataset.

**Non-canonical Cell Membrane Permeability Peptide (nc-CPP) Dataset:** The first web-accessible database for cyclic peptide membrane permeability, CycPeptMPDB [17], was employed to construct the nc-CPP dataset. This database collects data on 7,334 cyclic peptides, including structure and experimentally measured membrane permeability, sourced from 45 scientific publications and 2 pharmaceutical patents. CycPeptMPDB offers dual forms of data: membrane permeability physical quantities, such as LogP (an index of lipophilicity), and sequence information represented via HELM notation and monomeric structures. This dataset is particularly suited for regression analysis on membrane permeability, and facilities a refined estimation of cell-penetrating ability.

**Solubility (c-Sol) Dataset:** The PROSO-II dataset [18] forms the basis of our peptide solubility prediction task. This dataset stringently curated its data from pepcDB [19] and PDB [20] databases, and represents one of the largest datasets available for solubility model development and evaluation. The original dataset contains 829,299 sequences and their corresponding experimentally measured solubility. Following the preprocessing proposed in the study [21], we use the software CD-HIT to filter each set at a 30% similarity threshold in order to decrease the redundancy of the datasets. After this process, the number of positive and negative samples was 11307. After that, we use an amino acid sequence length of 100 as a threshold to filter peptides. The reason we did not use 50, as set in the above-mentioned c-CPP dataset, is that all amino acid sequences in that dataset are greater than 50. Ultimately, we selected 1,511 sequences, with 877 being positive samples and 634 being negative samples.

**Canonical Binding Affinity (c-Binding) Dataset:** We employ the workflow from the study [22] to construct a benchmark dataset for the binding affinity prediction task, and result in a total of 1806 protein-peptide pairs with annotated binding affinity. In this dataset, 16.832% of the peptide sequences contain non-canonical amino acids. However, most of the existing protein language models [23] and protein-peptide interaction models [22] cannot process these non-canonical amino acids. Such algorithms conventionally represent non-canonical amino acids with an "X" token, allowing the sequences to be fed into the models. For instance, the sequence for the peptide chain in the complex identified by PDB id 4UTN is notated as XGVLXEYGV [24], which contains non-canonical amino acids at the first and fifth positions. Therefore, this dataset retains the name "Canonical Binding" to ease the comparison with other models that are limited to processing only canonical amino acids.

**Non-Canonical Binding Affinity (nc-Binding) Dataset:** To verify the model's capability to model non-canonical amino acids, we construct the dataset of protein-binding peptides with the non-canonical amino acids using the same workflow as in the construction of the cBinding dataset, with a special focus on the peptides with non-canonical amino acids. The ultimately-obtained nc-Binding dataset contains 304 non-canonical peptides.

# Implementation Details

The computational experiments in this study were conducted on a high-performance Linux server equipped with 4 CPUs, each featuring 20 cores, and 4 Nvidia 3080 GPUs, each with 24 GB of memory. The system had 256 GB of RAM. The source code was implemented using Python 3.10.0, and the deep learning frameworks employed included PyTorch 1.10.1 and torch-geometric 2.0.3 for handling graph-based data structures and operations.

Atom-level features were extracted using a feature encoding function that captures various atomic properties, including atomic number, degree, formal charge, chiral tag, number of hydrogen atoms, hybridization state, aromaticity, and scaled mass. Bond-level features were derived by encoding bond types, conjugation status, ring membership, and stereochemical properties. Fragmentation of molecular structures was tailored for peptide data, employing a method that identifies and cuts specific bonds, resulting in molecular fragments. Each fragment's features were represented by concatenating MACCS key embeddings and pharmacophore property features, further supplemented by a rule-based edge generation process for fragment connectivity.

The training pipeline's detailed configurations and parameter values, including the specifics of feature extraction, are provided in the "Experimental Setup" section of the Supplementary Materials to ensure reproducibility. This comprehensive setup allows for replicating the experiments and validating the findings in diverse computational environments. The pretraining process consisted of two stages: in the first stage, nearly 8 million peptides were used, and the training took approximately 46.3 hours. In the second stage, involving fewer than 10,000 non-natural amino acids, the training was completed in 1.8 hours.

# Experimental Setup

The pre-training step configured the batch size as 512 to balance computational efficiency and memory constraints on our hardware. The pre-training process was conducted for 5 epochs due to the extensive size of the pre-training dataset and the rapid convergence observed in the masking task. The relatively straightforward nature of this task, coupled with the diminishing returns in validation loss improvement beyond the initial epochs, supported this decision. We monitored the validation loss during training and selected the checkpoint with the lowest loss for the optimal performance of downstream evaluations. The learning rate was initialized at 0.001, and the embedding dimensions for both atom-level and fragment-level representations were set to 300. The graph transformer architecture utilized in the model consisted of 5 layers, which provided sufficient depth to capture the intricate relationships in the molecular graph while maintaining computational tractability.

The experimental settings of the downstream tasks were tailored to the specific requirements of each task. The affinity prediction task was computationally intensive, and required 200 epochs to achieve convergence, while other tasks converged effectively within 100 epochs. Learning rates were adjusted based on the dataset and task complexity: a learning rate of 0.00001 was applied to the CPP (Cell Penetrating Peptide) prediction tasks on the c-CPP and nc-CPP datasets, reflecting the need for fine-grained optimization. For the remaining tasks, a learning rate of 0.00005 was employed. Across all downstream tasks, a batch size of 32 was consistently used to balance training stability and computational load. These configurations ensured robust performance while accommodating the varying demands of different tasks.

To the best of our knowledge, there is currently no representation model that is specifically tailored for both canonical and non-canonical peptides. In order to establish a comparative framework for the prediction of non-canonical peptide properties, we elected to use models from the small molecule domain as baseline comparators. We selected three types of pre-trained models in the comparative experiments, and these models harness different molecular information dimensions: one-dimension (1D), two-dimension (2D) and three-dimension (3D). Specifically, this study evaluated the following models. ChemBERTa-2 [25] is a 1D sequence-based language model trained on one of the largest small molecule datasets. MolCLR [26] trains a graph neural network through a 2D molecular contrastive learning strategy. Uni-Mol [27] is a universal 3D molecular representation learning framework. For tasks involving only canonical amino acids, We evaluated the above models as well as protein language models, such as ESM2 [28] and ProteinBert [29] for canonical peptide-based prediction tasks as comparison baselines. This study employed the largest version of ESM2 which features 48 transformer layers.

For peptide-specific properties such as cell penetrating ability and solubility, we used our PepLand model to extract peptide features. For properties involving protein-peptide interactions, such as affinity prediction, we utilized ESM2 to extract protein features. To ensure fairness in our evaluations, the protein feature extraction model remained consistent across all comparisons.

The performance of our proposed PepLand model was evaluated against these baseline models across all the designated datasets. The classification tasks were evaluated with the metrics such as accuracy (ACC), F1-score (F1), and the area under the receiver operating characteristic (AUC). Meanwhile, the regression prediction tasks were evaluated using the Pearson correlation coefficient (PCC) and Spearman correlation coefficient (SCC).

# Interpretability Analysis

To better understand how PepLand encodes peptide information, we performed a two-dimensional visualization of the learned features via t-SNE and compared them with features extracted from ChemBERTa (Supplementary Figure S9 and Supplementary Figure S10). As seen in these plots, PepLand’s embeddings exhibit clearer separations among data points, indicating that its learned representations are more discriminative. This is further supported by the clustering results over the t-SNE embeddings, where PepLand’s clusters are more cohesive and better aligned with the known peptide properties than those obtained from ChemBERTa.

To investigate why PepLand’s clusters appear more distinct, we analyzed the structural and physicochemical characteristics of peptides within each cluster. For example, as shown in the clustering results (Supplementary Figure S11), PepLand grouped peptides into distinct clusters with well-defined structural features:

- Cluster 17 predominantly contains peptides with hydrophobic side chains, including non-canonical amino acids with branched alkyl or aromatic substitutions. This cluster captures peptides with strong lipophilic properties, potentially suited for interactions with hydrophobic environments or specific protein-binding sites.
- Cluster 18 is primarily hydrophobic, with simpler, more compact substitutions compared to Cluster 17. The molecules appear to be more compact in three-dimensional space.
- Cluster 0 is characterized by a symmetric cyclic peptide core with a prominent large side chain, giving the molecules a bulkier appearance and higher perceived molecular weight. The design emphasizes rigidity and structural stability.

To investigate why PepLand’s clusters appear more distinct, we examined the distributions of key physicochemical properties (e.g., cLogP, TPSA, HBD, HBA, and molecular weight) within each cluster. Taking the “c_CPP” dataset in Supplementary Figure S12 as an example, PepLand’s clusters show relatively small intra-cluster variance yet larger inter-cluster variance. In other words, peptides grouped together by PepLand tend to share similar physicochemical characteristics, whereas peptides in different clusters show marked differences. This pattern strongly suggests that PepLand effectively captures the diverse structural and physicochemical attributes of peptides, thus providing more fine-grained and biologically meaningful feature representations.


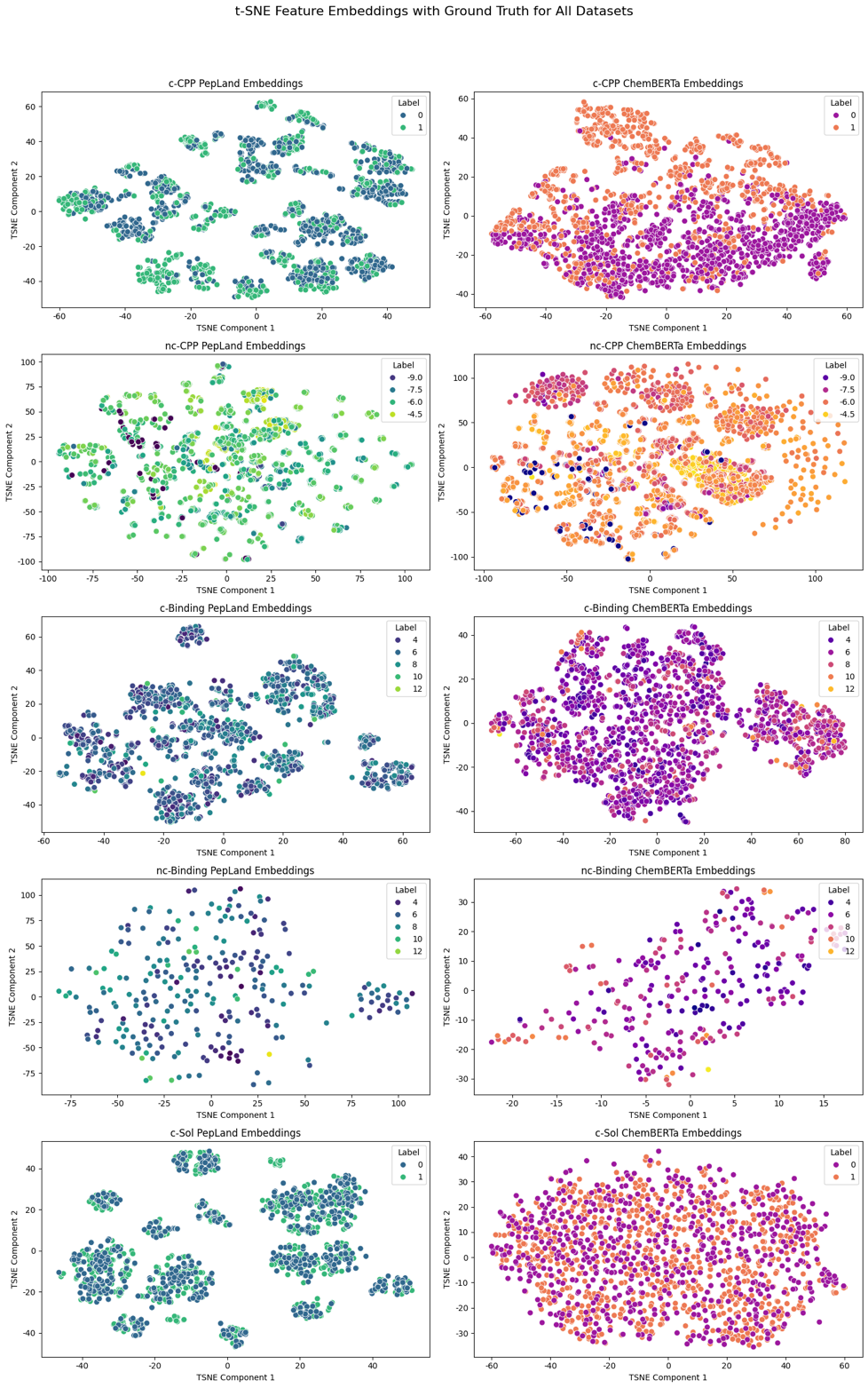


Supplementary Figure S9. t-SNE Feature Embeddings with Ground Truth for All Datasets. These plots display the 2D t-SNE projections of feature embeddings learned by PepLand and ChemBERTa across five datasets (c_CPP, nc_CPP, c_Binding, nc_Binding, c_Sol). Each point represents a peptide, and colors denote ground-truth labels. Clearer separations in the PepLand embeddings indicate its stronger discriminative power.


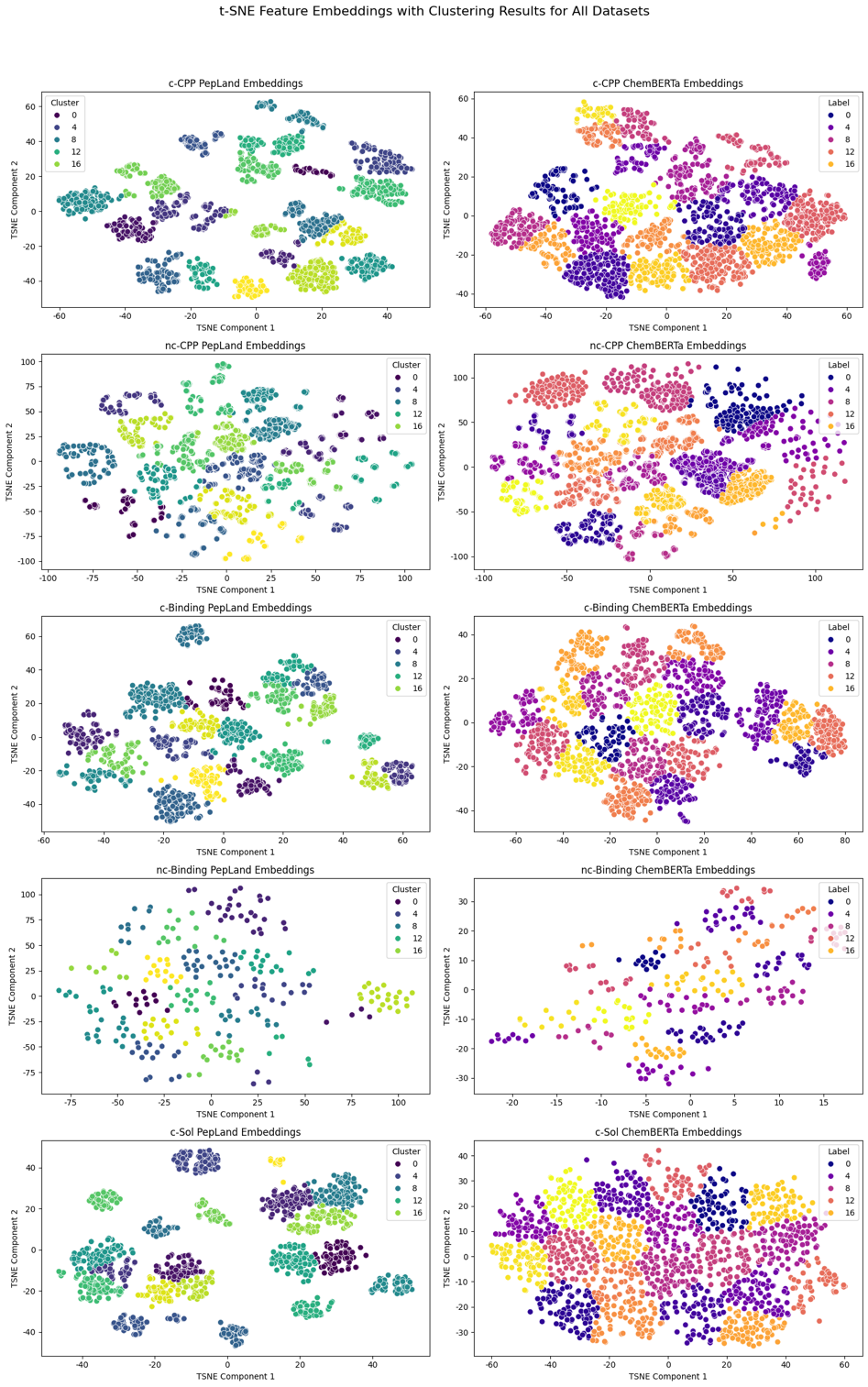


Supplementary Figure S10. t-SNE Feature Embeddings with Clustering Results for All Datasets. Using the same embeddings from Figure S9, these t-SNE plots show the clusters assigned by an unsupervised clustering algorithm. Different colors represent different clusters. PepLand’s clusters appear more coherent and distinct, suggesting more biologically meaningful feature representations.


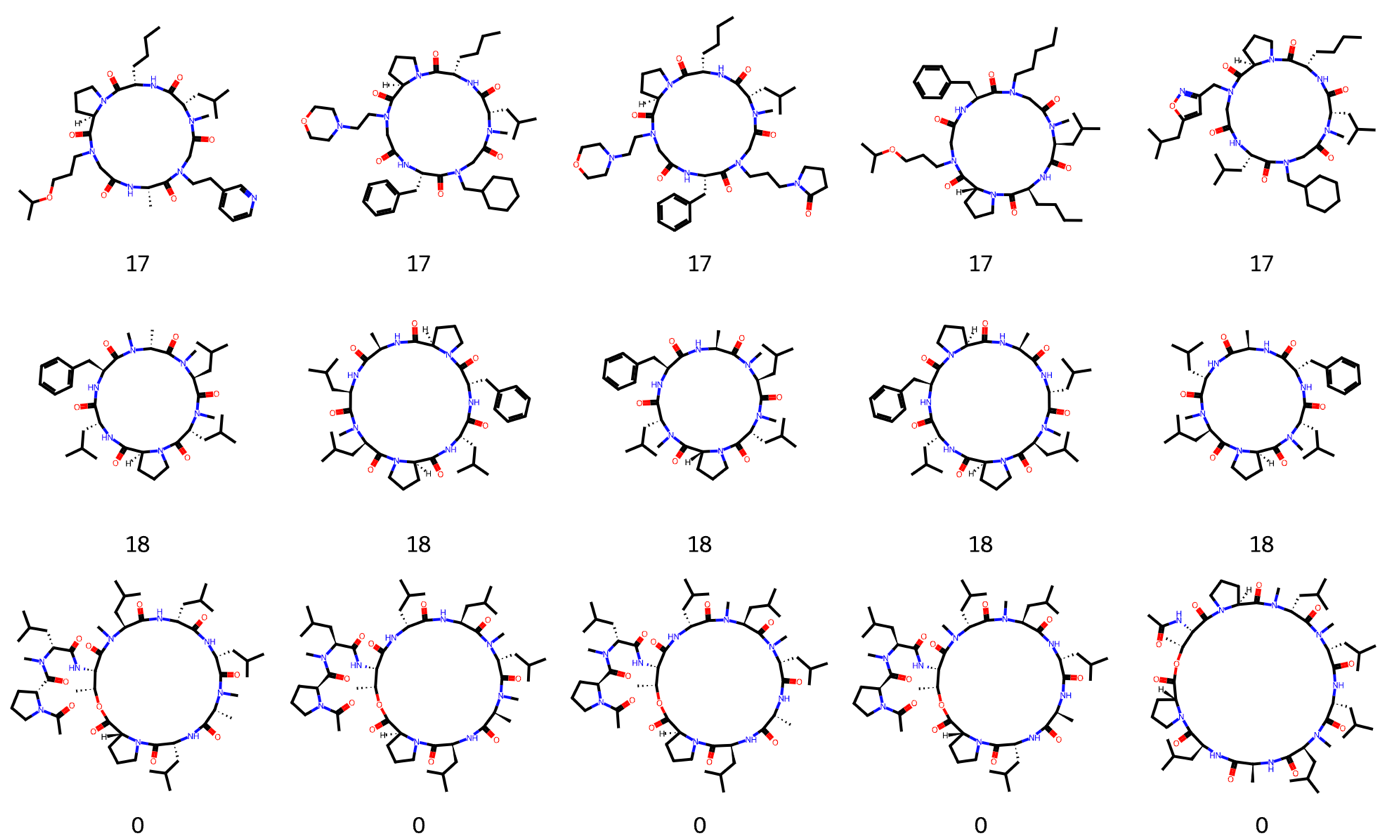


Supplementary Figure S11. Representative molecular structures from three selected clusters (Cluster 17, Cluster 18, and Cluster 0) obtained from the nc_CPP dataset. Clustering was performed using the KMeans algorithm with PepLand feature embeddings, dividing the data into 20 clusters. Each cluster highlights distinct structural characteristics.


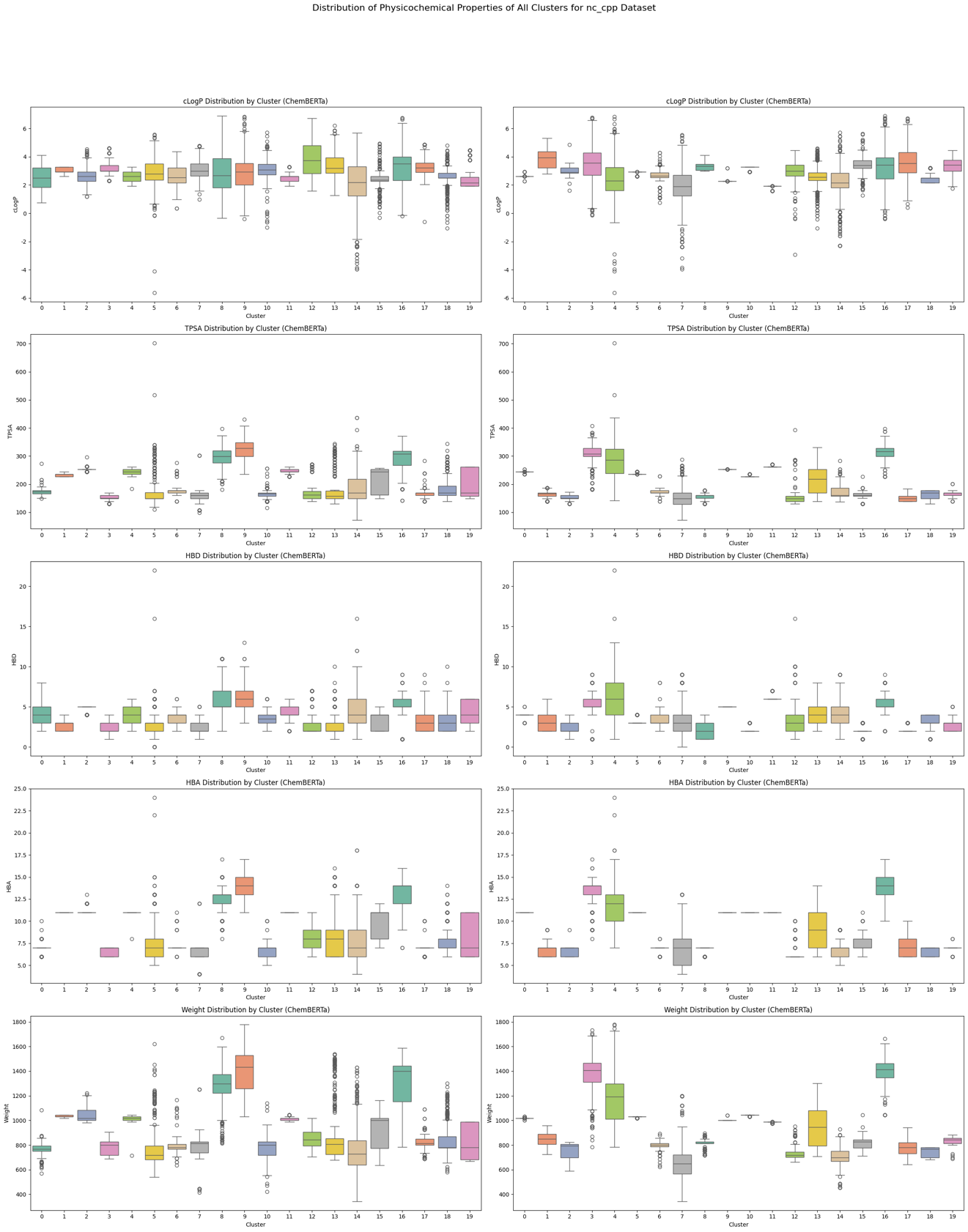


Supplementary Figure S11. Distribution of Physicochemical Properties for Each Cluster in the c_CPP Dataset. these box plots show how cLogP, TPSA, HBD, HBA, and molecular weight vary among clusters in the c_CPP dataset. By examining differences in these properties, one can see how each model’s features group peptides with shared characteristics.


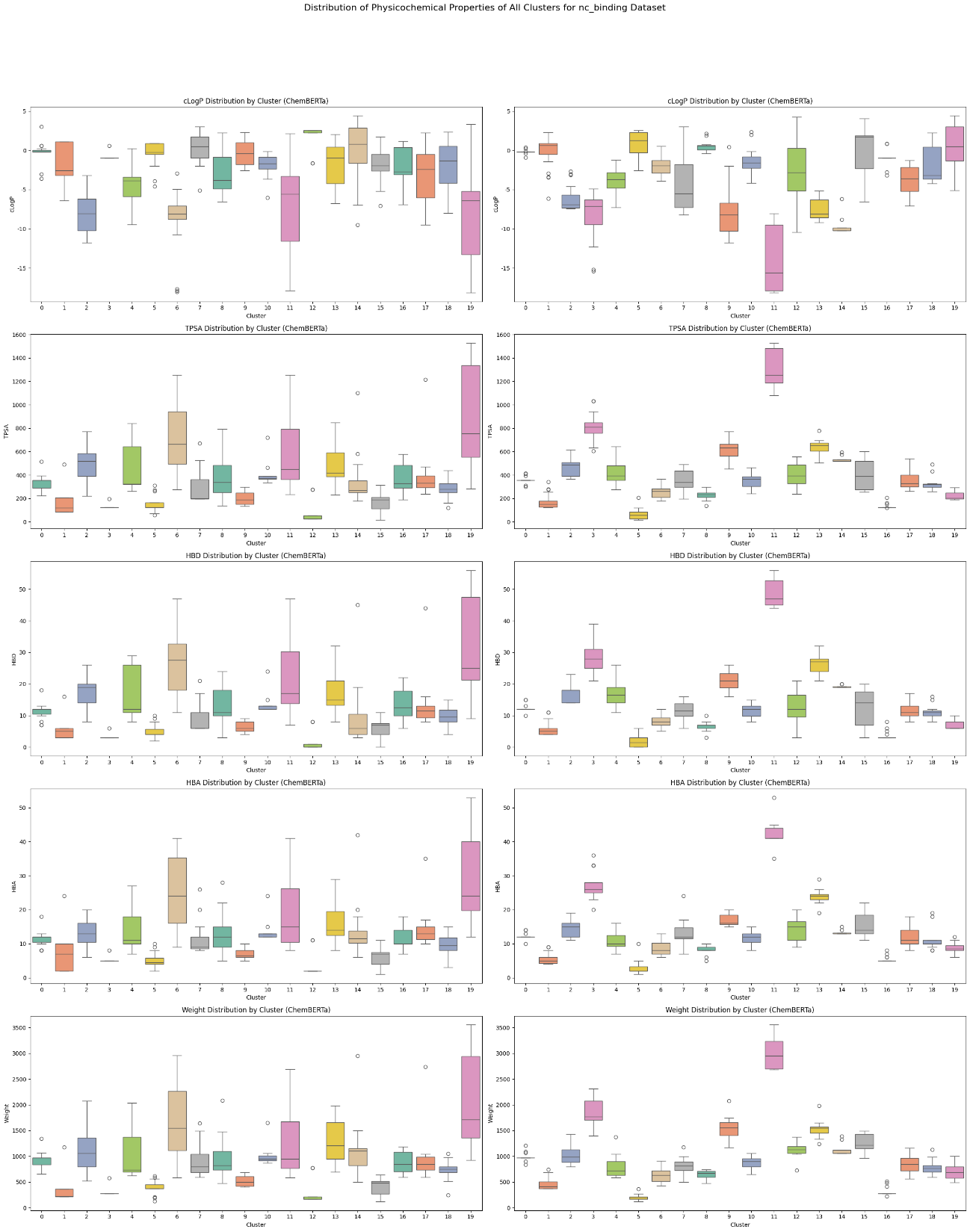


Supplementary Figure S12. Distribution of Physicochemical Properties for Each Cluster in the nc_Binding Dataset. Box plots compare cLogP, TPSA, HBD, HBA, and molecular weight across clusters identified by ChemBERTa and PepLand. Each cluster’s physicochemical profile is visualized, revealing how different subsets of peptides in the nc_Binding dataset exhibit distinct properties.

# Case Study 1: Binding Affinity Prediction of Cyclic Peptides

Supplementary Figure S13. The binding affinity prediction task is evaluated on two cases 1SMF and 5XCO. The histogram evaluates four models, i.e., HADDOCK, Rosetta, DeepPurpose, and PepLand. The horizontal axis gives the two cyclic peptides 1SMF and 5XCO, and the vertical axis gives the performance metric SCC.

In this case study, we focus on cyclic peptides owing to their significant pharmacological potential (Supplementary Figure S13). Additionally, cyclic peptides cannot be directly represented by amino acid sequences due to the involvement of various cyclization methods. We can consider cyclic peptides as peptides that contain non-canonical amino acids. We compare the PepLand’s predictions for binding affinity with three renowned open-source methods HADDOCK [30], Rosetta FlexDDG [31], and DeepPurpose [32]. HADDOCK is specialized for flexible docking in biomolecular complex modeling. Rosetta FlexDDG is a component of the comprehensive Rosetta macromolecular modeling suite, and excels in sampling conformational diversity to estimate interface binding affinity. DeepPurpose is a deep learning-based framework designed for predicting protein-ligand interactions.

Given the lack of established benchmarks for cyclic peptide binding affinity prediction, we curate a dataset encompassing cyclic peptides associated with two proteins from the SKEMPI 2.0 database [33]. These proteins are identifiable by the PDB IDs 5XCO and 1SMF, and are linked to the crystal structure of the human K-Ras G12D mutant with GDP and cyclic inhibitory peptides, and artificial trypsin inhibitor peptides, respectively. This dataset includes 10 peptides from the K-Ras G12D mutant and 6 from 1SMF. Each peptide is annotated with corresponding binding affinity. We exclude these peptides from our training dataset to eliminate the risk of data leakage, and use them only for post-training validation on two independent sets. While HADDOCK and Rosetta FlexDDG do not require training, the training process of DeepPurpose may have utilized the independent testing peptides.

Supplementary Figure S14 illustrates each data point’s predicted binding affinity across all tested methods. By inspecting each sample individually, one can clearly observe that PepLand’s predictions (red trendline and shaded confidence region) align more closely with the ground-truth binding affinities than those of the other models, underscoring the robustness and consistency of PepLand’s performance even in a small dataset scenario.

PepLand achieves exceptional prediction results on the 1SMF dataset, with an SCC of 0.829, and shows its potentially critical capability for virtual screening in drug discovery. Rosetta FlexDDG also exhibits comparable performance on 1SMF with SCC=0.770. However, HADDOCK and DeepPurpose lag behind, with the SCC values of only 0.265 and 0.436, respectively. Our PepLand model also attains the best SCC value of 0.718. Conversely, HADDOCK and DeepPurpose show poor performance with SCC of 0.190 and 0.260, respectively. Rosetta FlexDDG is unable to compute due to data limitations. In summary, PepLand significantly outperforms current Computer-Aided Drug Design (CADD) tools and other deep learning-based methods in predicting the binding affinity of cyclic peptides.


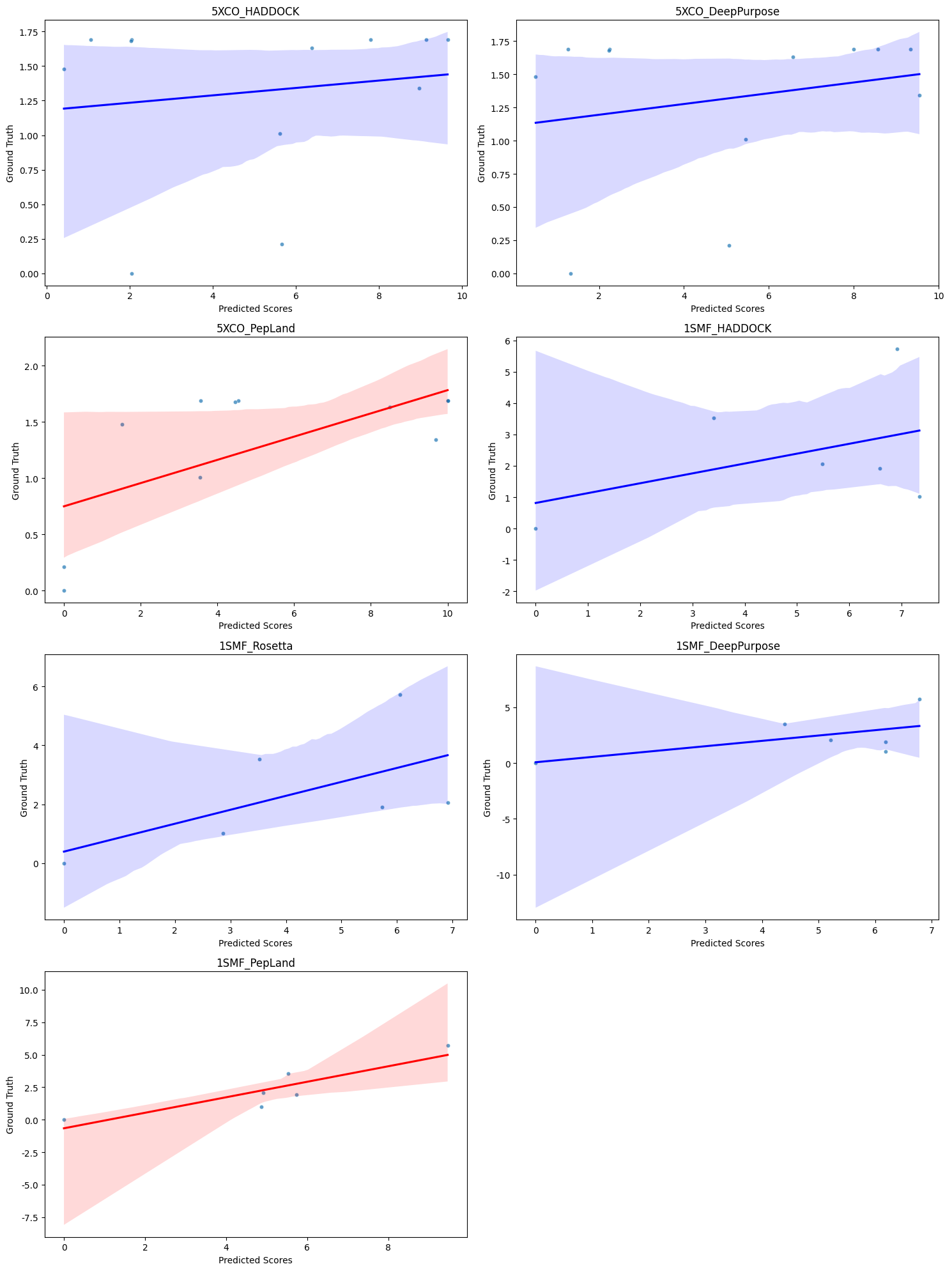


Supplementary Figure S14. Predicted versus experimentally measured $\Delta\Delta G$ for 5XCO and 1SMF using four methods: HADDOCK, DeepPurpose, Rosetta, and PepLand. Each point represents an individual peptide’s binding affinity, with the best-fit line indicating the overall correlation and the shaded region showing the 95% confidence interval. PepLand (red line) demonstrates a stronger linear relationship with the ground-truth values compared to the other methods (blue lines), highlighting its robust predictive performance even in a limited data setting. Rosetta does not work on the dataset 5XCO.

# Case Study 2: Peptide Synthesizability Prediction

Supplementary Figure S15. Evaluation of ECFP Fingerprints and PepLand for the peptide synthesizability prediction. The peptide synthesizability prediction model usually calculates four labels, i.e., Area, Height, Width, and Height-Width (H-W). The horizontal axis gives the two performance metrics MAE and R2, and the vertical axis gives the metric values. The symbols ↓ and ↑ indicate that a better performance is represented by a smaller MAE and a larger R2, respectively.

This case study investigates the chemical peptide synthesis, a process involving the step-by-step formation of amide bonds on a solid support. Mohapatra et al. proposed a deep learning approach based on the Extended Connectivity Fingerprints (ECFPs) for the peptide synthesizability prediction task [34]. The ECFP-based model outputs several metrics to describe the peptide synthesizability, including Area, Height, and Width. A critical metric highlighted in the study is the difference between the normalized versions of height and width (H-W). In the absence of a pre-trained model from [34], we train a new model using the code they provided and evaluated it against their benchmark dataset.

Supplementary Figure S15 assesses the features extracted by PepLand against the ECFP fingerprints. The integration of PepLand features results in a reduction of the metric Mean Absolute Error (MAE) in predicting the difference between peak height and width, from 0.160 to 0.138. The coefficient of determination (R2) is also improved from 0.644 to 0.746 by utilizing the PepLand features. The enhancement indicates that the pre-trained PepLand model excels in extracting features for the peptide synthesizability prediction task. The PepLand features also outperforms the ECFP features in the predictions of Height, Width, and Area.

# Case Study 3: Prediction Binding Affinity Prediction of Linear Peptides

To evaluate PepLand's effectiveness in real-world scenarios, we collected and tested it on 10 sets of linear peptide data from the SKEMPI 2.0 database [33]. The corresponding protein PDB IDs and dataset sizes are provided in Supplementary Table S2. However, identifying more real-world cases with sufficient data within SKEMPI remains challenging due to the scarcity of such datasets.

PepLand performs well on datasets with larger sample sizes, such as 3EQS and 3EQY, achieving Spearman correlation coefficients (SCC) above 0.9. While it also achieves SCCs of 1 on smaller datasets like 1GL0, 1GL1, and 3LNZ, these datasets each contain only 3 samples, and such results may not hold significant meaning due to the limited sample size. The model struggles with certain cases, such as 4CPA (SCC: 0.156) and 4J2L (SCC: 0), likely due to underrepresented peptide types in the training data, while moderate performance on cases like 1F47 (SCC: 0.516) and 1KNE (SCC: 0.7) suggests variability based on data complexity and sample size. These results highlight PepLand’s potential in practical applications while emphasizing the need for further testing on larger and more diverse datasets, as acknowledged in the Limitations section.

In summary, the experimental results demonstrate that the PepLand features are effect in peptide synthesizability prediction, and show the potential application in the field of peptide chemical synthesis.

Supplementary Table S2. 10 sets of linear peptide data collected from the SKEMPI 2.0 database. Num of Samples indicates the size of sets. SCC means the Spearman Correlation Coefficient for predicted score and ground truth.

| Case | Num of Samples | Peptide Type | SCC |
| --- | --- | --- | --- |
| 1F47 | 12 | Linear | 0.516 |
| 3EQS | 10 | Linear | 0.927 |
| 3EQY | 11 | Linear | 0.916 |
| 4CPA | 9 | Linear | 0.156 |
| 1GL0 | 3 | Linear | 1.000 |
| 1GL1 | 3 | Linear | 1.000 |
| 4J2L | 3 | Linear | 0.000 |
| 3LNZ | 3 | Linear | 1.000 |
| 3RF3 | 2 | Linear | 1.000 |
| 1KNE | 5 | Linear | 0.700 |

# References

1. Deng D, Lei Z, Hong X et al. Describe molecules by a heterogeneous graph neural network with transformer-like attention for supervised property predictions, ACS omega 2022;7:3713-3721.

2. Jiang Y, Jin S, Jin X et al. Pharmacophoric-constrained heterogeneous graph transformer model for molecular property prediction, Communications Chemistry 2023;6:60.

3. Zhang C, Song D, Huang C et al. Heterogeneous graph neural network. In: Proceedings of the 25th ACM SIGKDD international conference on knowledge discovery & data mining. 2019, p. 793-803.

4. deGruyter JN, Malins LR, Baran PS. Residue-specific peptide modification: a chemist’s guide, Biochemistry 2017;56:3863-3873.

5. Privalov PL, Gill SJ. Stability of protein structure and hydrophobic interaction, Advances in protein chemistry 1988;39:191-234.

6. Degen J, Wegscheid‐Gerlach C, Zaliani A et al. On the Art of Compiling and Using'Drug‐Like'Chemical Fragment Spaces, ChemMedChem: Chemistry Enabling Drug Discovery 2008;3:1503-1507.

7. Devlin J, Chang M-W, Lee K et al. Bert: Pre-training of deep bidirectional transformers for language understanding, arXiv preprint arXiv:1810.04805 2018.

8. Mikolov T, Chen K, Corrado G et al. Efficient estimation of word representations in vector space, arXiv preprint arXiv:1301.3781 2013.

9. Hu W, Liu B, Gomes J et al. Strategies for pre-training graph neural networks, arXiv preprint arXiv:1905.12265 2019.

10. Xia J, Zhu Y, Du Y et al. A survey of pretraining on graphs: Taxonomy, methods, and applications, arXiv preprint arXiv:2202.07893 2022.

11. UniProt C. UniProt: a hub for protein information, Nucleic acids research 2015;43:D204-D212.

12. Chen M, Radford A, Child R et al. Generative pretraining from pixels. 2020, p. 1691-1703 %@ 2640-3498. PMLR.

13. Agrawal P, Bhalla S, Usmani SS et al. CPPsite 2.0: a repository of experimentally validated cell-penetrating peptides, Nucleic acids research 2016;44:D1098-D1103.

14. Gautam A, Singh H, Tyagi A et al. CPPsite: a curated database of cell penetrating peptides, Database 2012;2012:bas015.

15. Deutsch EW, Lam H, Aebersold R. PeptideAtlas: a resource for target selection for emerging targeted proteomics workflows, EMBO reports 2008;9:429-434.

16. Fu L, Niu B, Zhu Z et al. CD-HIT: accelerated for clustering the next-generation sequencing data, Bioinformatics 2012;28:3150-3152.

17. Li J, Yanagisawa K, Sugita M et al. CycPeptMPDB: A Comprehensive Database of Membrane Permeability of Cyclic Peptides, Journal of Chemical Information and Modeling 2023;63:2240-2250.

18. Smialowski P, Doose G, Torkler P et al. PROSO II–a new method for protein solubility prediction, The FEBS journal 2012;279:2192-2200.

19. Kouranov A, Xie L, de la Cruz J et al. The RCSB PDB information portal for structural genomics, Nucleic Acids Res 2006;34:D302-305.

20. Burley SK, Bhikadiya C, Bi C et al. RCSB Protein Data Bank (RCSB.org): delivery of experimentally-determined PDB structures alongside one million computed structure models of proteins from artificial intelligence/machine learning, Nucleic Acids Res 2023;51:D488-d508.

21. Agostini F, Cirillo D, Livi CM et al. cc SOL omics: A webserver for solubility prediction of endogenous and heterologous expression in Escherichia coli, Bioinformatics 2014;30:2975-2977.

22. Lei Y, Li S, Liu Z et al. A deep-learning framework for multi-level peptide–protein interaction prediction, Nature communications 2021;12:5465.

23. Rives A, Meier J, Sercu T et al. Biological structure and function emerge from scaling unsupervised learning to 250 million protein sequences, Proceedings of the National Academy of Sciences 2021;118:e2016239118.

24. Roessler C, Nowak T, Pannek M et al. Chemical probing of the human sirtuin 5 active site reveals its substrate acyl specificity and peptide‐based inhibitors, Angewandte Chemie 2014;126:10904-10908.

25. Ahmad W, Simon E, Chithrananda S et al. Chemberta-2: Towards chemical foundation models, arXiv preprint arXiv:2209.01712 2022.

26. Wang Y, Wang J, Cao Z et al. MolCLR: Molecular contrastive learning of representations via graph neural networks. arXiv 2021, arXiv preprint arXiv:2102.10056.

27. Zhou G, Gao Z, Ding Q et al. Uni-Mol: a universal 3D molecular representation learning framework 2023.

28. Lin Z, Akin H, Rao R et al. Evolutionary-scale prediction of atomic-level protein structure with a language model, Science 2023;379:1123-1130.

29. Brandes N, Ofer D, Peleg Y et al. ProteinBERT: a universal deep-learning model of protein sequence and function, Bioinformatics 2022;38:2102-2110.

30. Dominguez C, Boelens R, Bonvin AMJJ. HADDOCK: a protein− protein docking approach based on biochemical or biophysical information, Journal of the American Chemical Society 2003;125:1731-1737.

31. Barlow KA, Ó Conchúir S, Thompson S et al. Flex ddG: Rosetta ensemble-based estimation of changes in protein–protein binding affinity upon mutation, The Journal of Physical Chemistry B 2018;122:5389-5399.

32. Huang K, Fu T, Glass LM et al. DeepPurpose: a deep learning library for drug–target interaction prediction, Bioinformatics 2020;36:5545-5547.

33. Jankauskaitė J, Jiménez-García B, Dapkūnas J et al. SKEMPI 2.0: an updated benchmark of changes in protein–protein binding energy, kinetics and thermodynamics upon mutation, Bioinformatics 2019;35:462-469

34. Mohapatra S, Hartrampf N, Poskus M et al. Deep learning for prediction and optimization of fast-flow peptide synthesis, ACS central science 2020;6:2277-2286.
